# Genome to single-cell characterization of the MAPK family in bighead carp (*Hypophthalmichthys nobilis*) spleen during *Aeromonas hydrophila* challenge

**DOI:** 10.3389/fimmu.2026.1777786

**Published:** 2026-04-13

**Authors:** Yang Mao, Xue Yu, Wenjing Wang, Jin Peng, Minao Wang, Jianyun Zhou, Defeng Li

**Affiliations:** 1 Clinical Medical Research Center, Xinqiao Hospital, Army Medical University, Chongqing, China; 2 Department of General Medicine, Changsha Township Central Hospital of Kaizhou District, Chongqing, China

**Keywords:** *Aeromonas hydrophila*, infection, mapk gene, single-cell RNA, WGCNA

## Abstract

**Introduction:**

Mitogen-activated protein kinases (MAPKs) are central to stress and innate immune signaling in fish, but their genomic composition and infection related transcriptional responses remain unclear in bighead carp (Hypophthalmichthys nobilis).

**Methods:**

We performed a comparative genomic survey across 24 species and characterized MAPK family members in bighead carp based on conserved motifs, gene structure, and synteny. Tissue expression profiling, transcriptome sequencing, qRT-PCR, weighted gene co-expression network analysis (WGCNA), and single-cell RNA sequencing were used to investigate MAPK expression and immune responses following Aeromonas hydrophila challenge. Cell-cell communication and pseudotime analyses were further applied to dissect infection responsive myeloid populations.

**Results:**

We identified 382 mapk sequences across 24 species, including 15 high confidence mapk genes in bighead carp, which were classified into the ERK, JNK, and p38 subfamilies. These genes were constitutively expressed across six tissues, with mapk12b and mapk14b showing relatively high expression in spleen and head kidney. Following A. hydrophila challenge, mapk6 and mapk11 were significantly upregulated, as confirmed by transcriptomic and qRT-PCR analyses. WGCNA further showed that multiple infection related modules were enriched in mapk family members. Single-cell RNA sequencing of 28,348 spleen cells identified 13 distinct cell types and revealed marked expansion and activation of Neutrophils-I cells and M1 macrophages, accompanied by increased mapk6/mapk11 expression and strong enrichment of interferon stimulated pathways. Cell-cell communication and pseudotime analyses further suggested that tnf-tnfrsf1b signaling and progressive upregulation of mapk6/mapk11 along myeloid lineages contribute to the early splenic antibacterial response.

**Conclusion:**

This study provides a genome to single-cell view of MAPK family organization and immune function in bighead carp, and identifies mapk6 and mapk11 as potential mediators of the early spleen response to bacterial infection.

## Highlights

- Defined the complete 15 mapk repertoires in bighead carp.
- Linked teleost duplication to extra mapk8/mapk12/mapk14 retention.
- Identified mapk6/mapk11 as infection associated network hubs.
- Localized mapk6/mapk11 induction to activated myeloid cells by scRNA-seq.
- Prioritized mapk6/mapk11 as key infection responsive candidates.

## 1 Introduction

The mitogen-activated protein kinase (MAPK) pathway is an evolutionarily conserved and functionally critical signaling system in eukaryotic cells (1, 2). It is ubiquitous in all eukaryotes, from yeast to humans (3). These kinases play a central role in regulating fundamental cellular activities. This includes cell growth, proliferation, differentiation, survival, development, gene expression, metabolism, cell movement, and responses to environmental stimuli (4–6). The MAPK signaling module typically consists of three sequentially acting kinases: MAPK kinase kinase (MKKK/MAP3K), MAPK kinase (MKK/MAP2K), and MAPK (7). These kinases sequentially activate downstream kinases through diphosphorylation of threonine and tyrosine residues. The MAPK protein activation circuit includes a conserved Thr-X-Tyr (TXY) diphosphorylation site. Based on the shared sequence of their TXY sites, the MAPK family can be divided into three major subfamilies, ERK (TEY), JNK (TPY), and p38 (TGY) (4, 8, 9), which preferentially respond to mitogenic signals (10), stress insults (11), and inflammatory stimuli, respectively, and thus exhibit distinct functional emphases (12).

Bony fishes have undergone a fish-specific genome duplication (FSGD), an event that provided the possibility of increased gene dosage and functional differentiation for multiple signaling pathways, including *mapk* (13–15). Therefore, multiple copies of *mapk* family members can be observed in the genomes of various fish species. For example, duplicate *mapk14* has been detected in species such as spotted bass and channel catfish, and double copies of *mapk8* and *mapk14* have been found in yellow catfish (14–16). This type of gene duplication is generally considered to facilitate more refined signal regulation or promote the emergence of new functions. In teleost fish, bacterial pathogens such as *Aeromonas hydrophila* can trigger strong innate immune responses and activate diverse immune regulatory pathways. Recent studies have shown that *A. hydrophila* infection induces complex innate immune regulatory mechanisms, including the activation of pattern-recognition receptors and the cGAS-STING signaling pathway in catfish species (17–19). Correspondingly, numerous studies have shown that the *mapk* family in fish can be induced by pathogen infection, inflammatory stimulation, and various abiotic stresses. The expression of *mapk6*, *mapk11* and *mapk14b* in Japanese turbot changes after infection with *Edwardsiella tarda* (20); *Mapk8a*, *mapk8b*, *mapk13*, and *mapk14b* in yellow catfish are enhanced after challenge with *A. hydrophila* (15); and upregulation of CiMEKK3 in grass carp is induced by *A. hydrophila* indicates that upstream kinases are also involved in the immune response in fish (21). Overall, these scattered studies point to the fact that the fish *mapk* does indeed participate in the response to biotic stresses, and that replicated members can often be mobilized into the immune context.

The advent of single-cell RNA sequencing (scRNA-seq) now allows cell-level dissection of immune heterogeneity in fish organs exposed to infection or environmental stress (22–25). While traditional bulk RNA sequencing (bulk RNA-seq) can reflect the overall expression trends of a sample, it cannot distinguish the differential responses of different cell populations under stress. scRNA-seq, however, can depict the cellular composition and transcriptional heterogeneity of tissues at the single-cell level and has been used in model fish such as zebrafish (*Danio rerio*) to identify various immune-related cell types and functional subsets under infection stress (26). Infection often induces intracellular interferon (IFN) responses, and IFN and its downstream ISGs play a crucial role in maintaining cellular homeostasis, resisting pathogens, and adapting to environmental changes (27, 28). However, current information on how different fish immune cell subsets simultaneously mobilize the *mapk* and IFN-related signals at the single-cell level under infection conditions remains limited, especially in non-model species with significant aquaculture value.

Bighead carp (*Hypophthalmichthys nobilis*), belonging to the Cyprinidae family of the Cypriniformes order, is a core member of the “Four Major Freshwater Fish” traditionally farmed in China, and is also an important fish species with significant economic value in global freshwater fisheries (29–31). Although the *mapk* gene family has been shown to be associated with various stress responses in many fish species, systematic research on the *mapk* gene family of bighead carp, an important freshwater aquaculture fish in China, remains very limited. In particular, there is a lack of detailed functional analysis of bighead carp *mapk* genes at the single-cell level, and research on their pseudo-temporal expression patterns under biological stress conditions, specifically, how cell types respond. A deep understanding of the structure, evolution, function, and stress response patterns of the bighead carp *mapk* in different cells is crucial for revealing the molecular mechanisms by which bighead carp adapt to environmental changes and resist disease. This will also provide a new theoretical basis and potential biomarkers for healthy bighead carp aquaculture. This study aims to comprehensively identify the members of the *mapk* gene family in the bighead carp genome and analyze their gene structure and phylogenetic relationships. More importantly, we will focus on exploring the expression patterns and temporal variations of different *mapk* members in specific cell types under the stress of biological infection, to elucidate the functional mechanisms of the *mapk* gene family in the immune defense against diseases in teleost fish. The findings of this study will contribute to the deep understanding of the *mapk* gene evolution, infected adaption strategy and molecular mechanisms of spleen remodeling in fish.

## 2 Materials and methods

### 2.1 Gene acquisition and identification

In summary, we analyzed genomes from 24 species spanning the vertebrate lineage, including a tunicate outgroup, fishes, amphibians, reptiles, birds, and mammals (**Supplementary File 1****,** **Supplementary Table 1**). First, all publicly available sequences were retrieved from the National Center for Biotechnology Information (NCBI). To identify additional MAPK candidates, custom databases were constructed for each genome and subsequently queried using tBLASTn (v2.6.0+, E-value ≤ 1e−5) for protein-to-nucleotide alignment, with human (*Homo sapiens*) and zebrafish (*Danio rerio*) MAPK proteins serving as query sequences (**Supplementary File 1**, **Supplementary Table 2**). A Perl script was then used to parse the resulting alignments and determine the best matches. For each best-match locus, genomic regions were extended 5–10 kbp upstream and downstream to capture potential full-length gene sequences, followed by GeneWise analysis to predict complete *mapk* gene structures (32). The following parameters were determined using Euk-mPLoc 2.0 (http://www.csbio.sjtu.edu.cn/bioinf/euk-multi-2/), including amino acid sequence length, molecular weight (MW) and isoelectric point (pI) (33). Subcellular localization of *mapk* was predicted using WoLF PSORT (https://wolfpsort.hgc.jp/) (34).

### 2.2 Mapk gene structure characterization and conserved motifs

To identify conserved motifs within *mapks*, all high-confidence *mapk* sequences were submitted to MEME (v5.5.8; https://meme-suite.org/meme/tools/meme) using the following parameters: an unrestricted number of repetitions, a maximum of 10 motifs, and motif lengths ranging from 6 to 50 amino acid residues (35). Structural features of the *mapk* sequences, including coding sequences (CDS), exon-intron architectures, and untranslated regions (UTRs), were systematically collected and extracted using a custom Perl script and subsequently visualized using GSDS (http://gsds.cbi.pku.edu.cn/) (36). Finally, TBtools was utilized to integrate and simultaneously visualize phylogenetic relationships, gene structures, and conserved motif patterns across the *mapk* gene family (37).

### 2.3 Sequence alignment and phylogenetic analyses

Phylogenetic analysis was performed utilizing *mapk* sequences. Initially, multi codon sequence alignment was conducted using MAFFT (v7.526) with the G-INS-I precision strategy (38). Subsequently, the aligned sequences underwent conservative trimming in Gblocks (v0.91b) employing the following parameters, a minimum of 75 conserved flanking sites, a maximum of 50 consecutive non-conserved sites, a minimum block length of 50 for conserved regions, and allowing gaps in all positions (39). Following this step, the ModelFinder module within IQ-TREE (v3.0.1) was utilized to determine the optimal substitution model based on Bayesian Information Criterion (BIC) (40). A maximum likelihood (ML) phylogenetic tree was then constructed using the GTR+F+R8 model, with branch support evaluated through 1,000 bootstrap resamples. Finally, the resulting phylogenetic tree was visualized and annotated using iTOL (41).

### 2.4 Synteny analysis and conservation identification

To evaluate the conservation and orthologous relationships of *mapk* genes, we initially retrieved flanking gene sets located upstream and downstream of the *mapk* locus through Ensembl (https://www.ensembl.org/biomart/martview/). Subsequently, synteny was cross-validated in Genomicus (https://www.genomicus.bio.ens.psl.eu), utilizing the human *mapk* locus as an anchor point (42). Candidate orthologous genes were identified and compiled within a 50 Mb window encompassing both the human and zebrafish *mapk* loci; nucleotide sequence alignments were then conducted on the identified syntenic genes. Finally, Perl scripts were employed to parse and assess the quality of tBLASTn alignments, thereby identifying optimal matches to enhance annotation accuracy.

### 2.5 Animals, infection model, and study design

**Figure 1:**
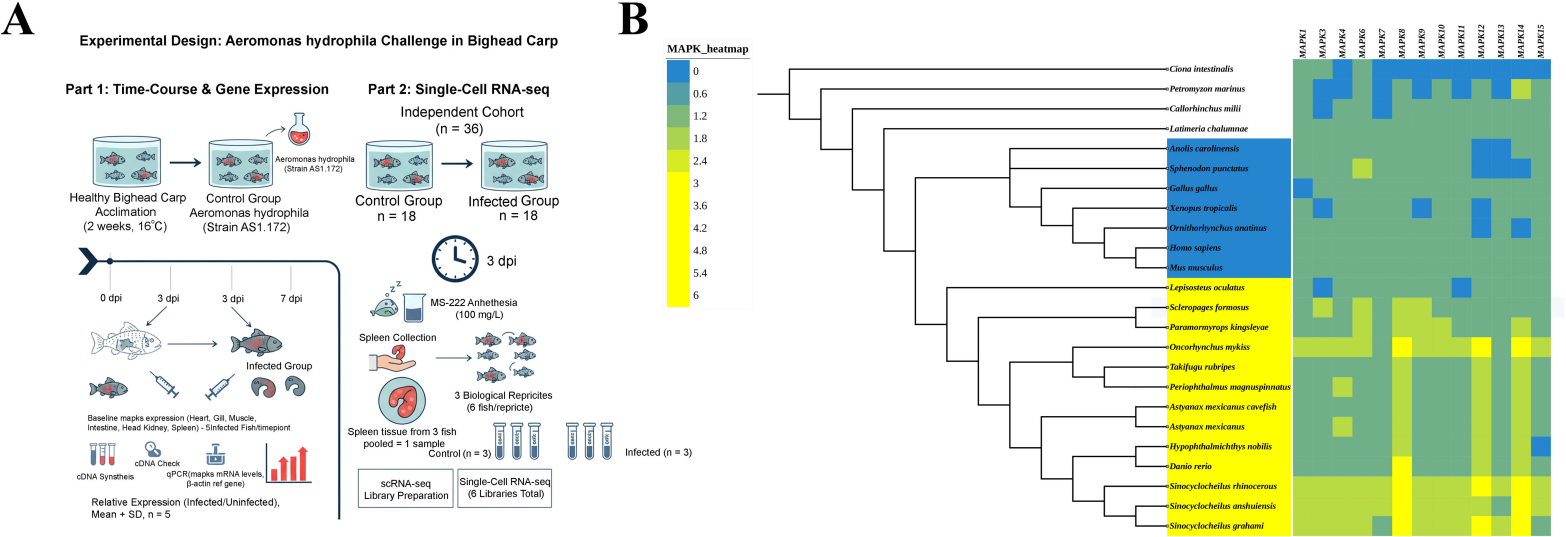
Study workflow and *mapk* repertoire across vertebrates. **(A)** Workflow of the study. **(B)** *Mapk* gene members in selected vertebrate species.

The experimental design is illustrated in **Figure 1A**. Healthy juvenile bighead carp (~100 g) were obtained from Kangyu Agriculture Co., Ltd. (Chongqing, China) and maintained at the Clinical Research Center in an aerated recirculating system at 16 °C under a 12:12 h light: dark cycle; before experimentation, fish were acclimated for two weeks and fed a commercial diet (Guangdong, China) twice daily, and no clinical signs were observed. *Aeromonas hydrophila* (strain AS1.172) was isolated from diseased grass carp at the College of Oceanography, South China Agricultural University (Guangzhou, China), and the isolate was confirmed by standard biochemical assays; in naturally infected fish, typical features include skin and fin haemorrhage, gill congestion, and visceral haemorrhage.

For the challenge experiment, thirty bighead carp were randomly assigned to control and infected groups. To establish a baseline tissue profile, total RNA was extracted from the heart, gill, muscle, intestine, head kidney, and spleen of five control fish to assess *mapks* expression. For the time course analysis, total RNA was extracted from the spleen and head kidney of five infected fish at 1-, 3-, and 7-days post-infection (dpi) (43). cDNA was synthesized through reverse transcription; To capture the peak innate response, single-cell sampling was conducted at 3 dpi in an independent cohort of 36 fish (18 for each group) with three biological replicates per group (six fish per replicate). At this time point, fish were anesthetized with MS-222 (tricaine methanesulfonate; 100 mg/L), buffered with sodium bicarbonate (NaHCO_3_; MS-222: NaHCO_3_ = 1:2, w/w) to a final pH of 7.0–7.5 (verified using pH strips), after which spleens were collected. Where applicable, fish were euthanized by MS-222 overdose (400 mg/L, buffered with NaHCO_3_ to pH 7.0–7.5) prior to tissue collection. For each replicate sample, spleen tissue from three randomly selected fish was pooled together to create one sample set for analysis. This process yielded six scRNA-seq libraries for three per group. All animal studies received approval from the Animal Experimentation Ethics Committee of Army Medical University (No. AMUWEC20255429, **Supplementary File 2**).

### 2.6 RNA extraction and qRT-PCR

Fish were euthanized prior to tissue collection using buffered MS-222 prepared under the same conditions and procedure described in Section 2.5. Total RNA was extracted from six tissue samples (gill, spleen, intestine, muscle, head kidney, and heart) employing a combination of TRIzol (Invitrogen) and the RNAeasy Mini kit (Qiagen, Shanghai, China), as previously described (44). The relative mRNA expression levels of *mapks* in various tissues were assessed through qPCR utilizing an ABI 7500 instrument (Applied Biosystems, Foster City, CA, USA). The housekeeping gene *β-actin* served as the reference gene. Specific qPCR primers for both *β-actin* and *mapks* are detailed in **Supplementary File 1**, **Supplementary Table 3** and were designed using Primer5 software. The relative expression levels of *mapks* were calculated according to the 2^-△△Ct^ method (45). All data are shown as mean ± SD of five biological replicates (n = 5), with three technical qRT-PCR replicates per sample.

### 2.7 External cohort validation

The *mapk* expression pattern in zebrafish under *A. hydrophila* infection was investigated using microarray dataset to provide an external, cross-species reference for assessing the conservation of infection induced *mapk* responses and to facilitate comparison with the bighead carp results. Raw NCBI data were sourced from GSE57952 (46), and differentially expressed genes (DEGs) were identified with the Limma R package (version 3.48.3) (47), selecting those with *p* < 0.05 and absolute log2FC (fold change) values > 1 (48). DEG enrichment analysis was conducted using the clusterProfiler package. To further explore gene modules related to bacterial infection, a scale-free network was constructed using the weighted correlation network analysis (WGCNA) R package, with the height threshold set to 0.25 (49).

### 2.8 Tissue dissociation and cell purification

Spleens were placed in ice-cold PBS (0.04% BSA), minced, and incubated in dissociation buffer (collagenase IV 3.5 mg/mL, papain 2 mg/mL, DNase I 120 U/mL) at 37 °C, 100 rpm for 20 min with gentle trituration. The reaction was stopped with PBS containing 10% FBS. Cell suspensions were passed through 70 µm and 40 µm strainers and centrifuged at 4 °C, 300 g for 5 min. Red blood cells were lysed with 1× RBC lysis buffer (2–5 min on ice), followed by immediate centrifugation and resuspension in PBS (0.04% BSA) (50). Viability was assessed by trypan blue (target ≥85%); a dead-cell removal kit (Miltenyi, 130-090-101) was used only if required. Cell concentration was adjusted to 700–1,200 cells/µL.

### 2.9 Single-cell RNA sequencing and library preparation

Single-cell libraries were prepared with 10x Genomics Chromium Single Cell 3′ v3.1 following the manufacturer’s instructions (GEM generation, reverse transcription and library construction). Libraries were sequenced on an Illumina NovaSeq 6000 (PE150) with a target depth ≥30,000 reads per cell (minimum ≥20,000). Cell Ranger v7.2.0 was used for demultiplexing, barcode/UMI processing and 3′ counting, aligning to a bighead carp reference built from genome FASTA and GTF.

### 2.10 Bioinformatic analysis of scRNA-seq data

Cell Ranger outputs were processed in Seurat v4.4.0 (51). Cells were retained if UMI ≤20,000, detected genes 500-4,000 and mitochondrial reads ≤15%. Data were log-normalized; 3,000 highly variable genes were selected. Datasets were integrated by canonical correlation analysis to mitigate batch effects, a graph was built on the first 35 principal components, clustering used an SNN approach (resolution = 0.5), and UMAP was used for visualization. Differential genes were identified with Wilcoxon tests in FindAllMarkers (|log2FC| > 0.25, BH-FDR < 0.05, pct > 10%) for cluster annotation by known markers. Within each cell type, FindMarkers compared control vs infected using the same thresholds, followed by GO and KEGG enrichment. Cell–cell communication was inferred with CellChat v2 (CellChatDB.zebrafish, default settings) (52). Myeloid trajectories were reconstructed with Monocle2 in DDRTree method (53).

### 2.11 Statistical analysis

One-way ANOVA followed by Duncan’s multiple range test was used to compare the cross sectional area of cells across time points. Differences in cell number between the control and infected groups were assessed with a two-tailed Student’s t-test. Data are presented as mean ± SEM, and *p* < 0.05 was considered statistically significant.

## 3 Results

### 3.1 Identification and analysis of bighead carp homolog mapk genes

A total of 382 *mapk* gene sequences were obtained from 24 species, among which 15 sequences from bighead carp genome were identified as encoding MAPK proteins. (**Figures 1B**, **Supplementary File 1**, **Supplementary Table 4**). Analysis of the physicochemical properties of HnMAPK (*Hypophthalmichthys nobilis mapk*) proteins showed that their lengths of the HnMAPK proteins ranged from 225 aa (HnMAPK14b) to 728 aa (HnMAPK6). The molecular weights of the HnMAPK proteins varied from 55.44 to 180.39 kDa, and the predicted pI values ranged from 4.87–5.11. All protein pI values were below 7, indicating that the MAPK proteins were more acidic. In addition, all MAPK proteins were predicted to be localized to the cytoplasm and nucleus (**Supplementary File 1**, **Supplementary Table 5**). In conjunction with the *mapk* sequences of other teleosts, these results suggest that teleosts with FSGD possess two *mapk8*/*mapk12/mapk14* isotypes (*mapk8a*, *mapk8b*, *mapk12a*, and *mapk12b* et al.). Furthermore, the lobe-finned fish coelacanth (*Latimeria chalumnae*), basal non-teleost ray-finned fish spotted gar (*Lepisosteus oculatus*), and cartilaginous fish elephant shark (*Callorhynchus milii*), which did not have extra genome duplication, possessed around 13 *mapk* genes, as expected. Notably, two copies of the *mapk6* and *mapk9* gene were detected in *Scleropages formosus* and *Paramormyrops kingsleyae*, despite not shown in other diploid teleosts.

### 3.2 Analysis of the mapk gene structure and motifs in bighead carp

**Figure 2:**
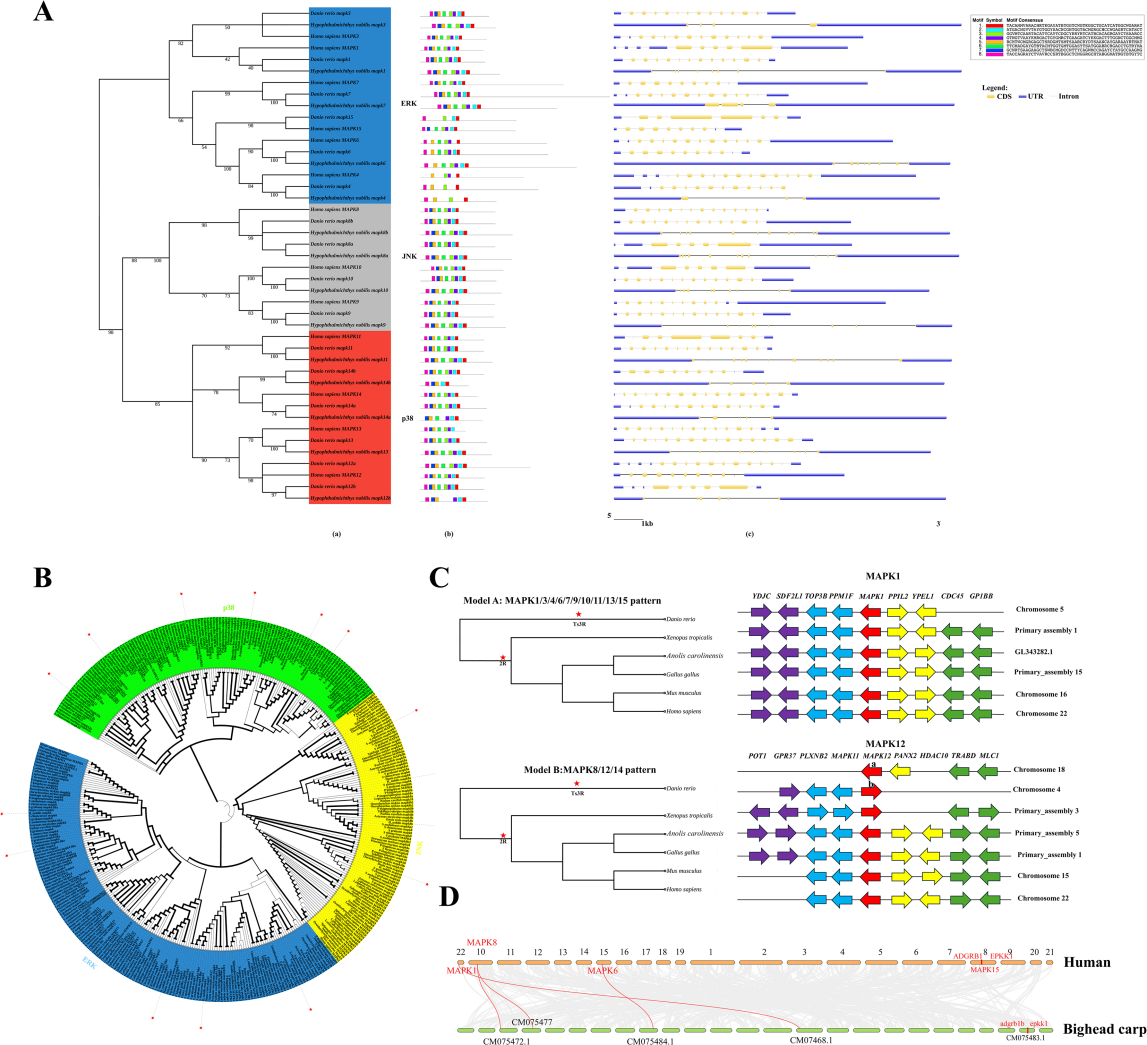
Gene identification among vertebrates. **(A)** The phylogenetic relationships, gene structures, and conserved motifs of 43 *mapk* genes in human, zebrafish and bighead carp. **(a)**: Using full-length nucleotide sequences from 43 *mapk* genes and 1,000 bootstrap test replicates, an unrooted maximum likelihood phylogenetic tree was constructed in IQtree. **(b)**: Conservation of motifs in the *mapk* genes. The potential motifs are represented by different colored boxes. **(c)**: *Mapk* gene exon/intron organization. Exons are represented by yellow boxes, and introns are represented by black lines of the same length. Blue boxes indicate the regions up-stream and downstream of the *mapk* genes (Untranslated regions, UTR). The scale at the bottom indicates the lengths of exons. See **Supplementary Figure 1**, **S2** in the **Supplementary Materials** for more information. **(B)** Maximum likelihood phylogram of relationships between *mapk* sequences from 23 representative vertebrates. The coding sequences of *mapk* genes were used to conduct phylogenetic reconstructions. The outgroup was a *Ciona intestinalis mapk* sequence. The blue background represents ERK, the green background represents p38 and the yellow background represents JNK. The *mapk* of bighead carp are marked with red asterisks. **(C)** Synteny analysis of the *mapk* genes associated to the phylogenetic tree. Orthologous genes flanking *mapk* show two patterns and highly conserved synteny among the vertebrates examined. Orthologous genes are shown in the same color arrows and identified from these synteny analyses in Genomicus mapper. Arrows indicate the gene direction relative to *mapk*, and red stars are mean the genome duplication events. **(D)** The synteny of *mapk* members between human and bighead carp. Notably, *mapk15* was not detected on scaffold CM075483.1 in bighead carp.

Gene structure is an important evolutionary feature of a gene and provides important clues to its functional diversification. Thus, the exon-intron and motif organization of these *mapk* genes were further analyzed (**Figure 2A**). The results revealed that the *mapk* of bighead carp contains approximately 5–10 exons, a 5’ region longer than the 3’ region in the UTR (**Supplementary File 3**, **Supplementary Figure 1**), and 8 motifs (**Supplementary File 3**, **Supplementary Figure 2**). Genes in the same subfamily showed similar gene structures, such as *mapk4* and *mapk7*, which possessed fewer motifs than other *mapks* (**Figure 2A**). This result is consistent with the conclusions of previous studies that the bighead carp 15 *mapk* genes can be broadly divided into three subgroups, ERK, p38 and JNK. In contrast, some variations between the UTR suggest that differential regulatory processes might be involved, although they are highly conserved in vertebrates.

### 3.3 Evolution of the mapk gene inferred from phylogenetic analysis

To better understand the relationships between *mapk* genes in vertebrates, we used ML to build a robust phylogenetic tree from 382 predicted *mapk* sequences, with the *Ciona intestinalis mapk* gene serving as the outgroup (**Figure 2B**). These 382 genes were classified into three distinct subfamilies based on their well-supported phylogenetic topologies (*mapk1/3/4/6/7/15, mapk11/12/13/14* and *mapk8/9/10*). Phylogenetic analyses suggest an apparent loss of *mapk15* in bighead carp; however, assembly quality cannot be excluded as a confounder, as *mapk15* is recovered in related carp species. All *mapk* genes from the tetrapod formed a sister group with those from teleosts, indicating consistency of the topology. It is worth noting that tuatara (*Sphenodon punctatus*) retained two *mapk6* genes, while lost *mapk12*, *mapk13* and *mapk14*.

### 3.4 Synteny analysis

Conserved synteny regions defined by neighborhoods linking two or more orthologous genes on a single chromosome or a chromosomal fragment in each of two or more different species provide critical information on how genes evolve (54). The genome context of the homologous genes was analyzed to verify the evolution inferred from phylogenetic analysis. In general, the bighead carp *mapk* gene family has two syntenic patterns, *mapk1/3/4/6/7/9/10/11/13* and *mapk8/12/14* (**Figure 2C**). In addition, all *mapk* gene families in this study showed remarkable synteny conservation among vertebrates, although the teleost clade seemed to be less conserved than the tetrapod clade (**Figure 2D**). From the syntenic results, the generation of additional *mapk* copies via tandem duplication in multiple species was confirmed. (**Supplementary File 1**, **Supplementary Table 6**, **S7**; **Supplementary Figure 2**).

### 3.5 Constitutive expression analysis of bighead carp mapk genes

**Figure 3:**
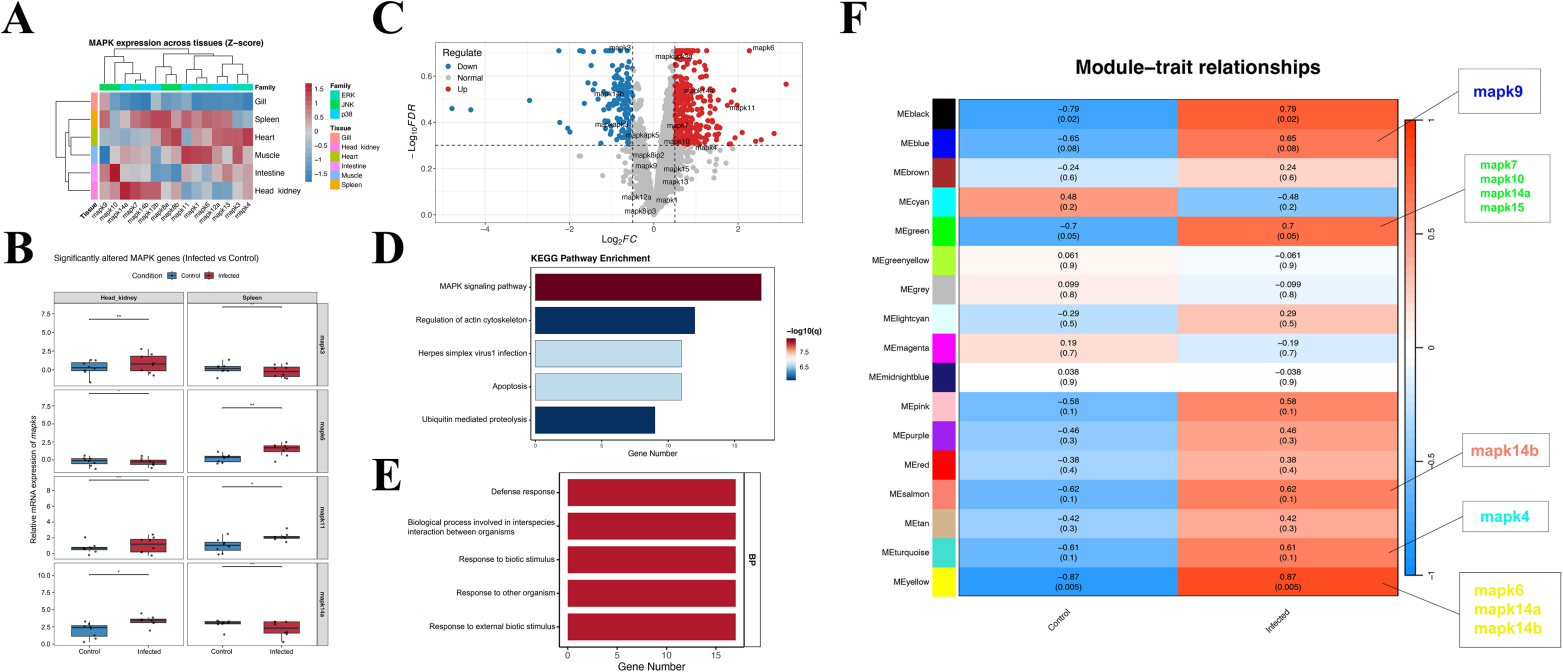
*Mapk* tissue profiles and infection responsive networks. **(A)** Baseline *mapk* family expression across tissues. **(B)** RT-qPCR boxplots for selected *mapks* in head kidney and spleen comparing control and infected fish. A lower ΔCt indicates higher expression; asterisks denote significance. **(C)** Volcano plot of spleen bulk RNA-seq (infected vs control) with *mapk* genes highlighted. **(D)** Top 5 KEGG enrichment of differentially expressed genes. **(E)** GO Biological Process enrichment emphasizing host-defense and biotic stimulus responses. **(F)** WGCNA module trait relationships for control and infected groups. Colors reflect correlation (red = positive, blue = negative); cells show correlation (FDR). Labels on the right indicate modules containing representative *mapk* genes. (Student’s t-test; *p < 0.05; **p < 0.01; ***p < 0.001).

The transcription levels of the 15 genes identified in the bighead carp genome were investigated in six tissues obtained from bighead carp (**Figure 3A**). According to the results of qRT-PCR, all *mapk* genes were constitutively transcribed in all six studied tissues. In general, all genes showed different transcription patterns, with the highest expression in the spleen/heart/head kidney and lowest expression in the gills. Notably, immune related tissues (spleen and head kidney) exhibited overall higher basal *mapk* expression than non-immune tissues, suggesting a stronger requirement for MAPK signaling under immune surveillance at homeostasis. Furthermore, differences in the transcription levels of various *mapk* genes have been discovered. In immune organs, such as the head kidney and spleen, *mapk12b/mapk14b* frequently showed higher transcript levels than other *mapk* genes. In contrast, *mapk4*, *mapk8b*, and *mapk10* had higher transcription levels in homeostasis tissues and organs, such as the heart and intestine (**Figure 3A**), where apoptosis is common. The *mapk14a* gene had the lowest levels of constitutive transcription, except in the head kidney. This constitutive tissue atlas provided an initial prioritization of infection relevant candidates that were further evaluated under *A. hydrophila* challenge and at single-cell resolution.

### 3.6 Expression patterns of mapk genes after infection in fish

To analyze the role of the *mapk* gene in response to *A. hydrophila* infection in fish, we combined GEO data and qRT-PCR analysis to evaluate the expression level of the *mapk* in zebrafish (GSE57952) and bighead carp (**Figure 3B, C**; **Supplementary File 3**, **Supplementary Figure 3**). Overall, *mapk6* and *mapk11* were significantly upregulated under *A. hydrophila* stress, while *mapk3* was downregulated after infection, but with slight differences in the two fish. The consistent induction of *mapk6* and *mapk11* across two cypriniform fishes highlighted these genes as robust infection associated candidates and motivated their subsequent validation at the single-cell level. The enrichment results showed that the *mapk* gene was mainly enriched in the MAPK signaling pathway and Defense response biology process in the experimental group (**Figures 3D, E**). In addition, the WGCNA results showed that the yellow module was most positively correlated with the occurrence of infection. In contrast, the cyan module was most negatively correlated with the occurrence of infection, according to the eigengene adjacency heatmap (**Figure 3F**). This WGCNA analysis revealed that *mapks* were enriched in the different modules, rather than isolated DEGs, related to infection occurrence.

### 3.7 Spleen single-cell transcriptome profiles of bighead carp under biotic stress

Single-cell transcriptome sequencing of bighead carp spleens was performed using the 10× genomics platform. The obtained scRNA-seq sequences were then aligned to the bighead carp genome. Sequencing and mapping quality were high, and after standard Seurat filtering we retained 28,348 high quality spleen cells from six samples (three replicates per group), which were sufficient for downstream clustering and comparative analyses (**Supplementary File 1**, **Supplementary Tables 8**-**S9**) (55). To further investigate the effects of *A. hydrophila* on bighead carp spleens, we selected 3,000 highly variable genes for principal component analysis (PCA). After PCA, cluster analysis, and UMAP downscaling, we clearly identified 13 cell clusters (**Supplementary File 3**, **Supplementary Figure 4A**). The top 10 DEGs with the highest enrichment in each cell cluster were visualized (**Supplementary File 3**, **Supplementary Figure 4B**). According to previous reports (23), these 13 cell clusters were classified into 13 cell types, including epithelial cells, hepatocytes, 9 immune cells, hematopoietic stem cells (HSCs), and proliferating cells. A detailed summary of the canonical marker genes used for annotation of each cell population has been provided in **Supplementary File 1**, **Supplementary Table 11**. The top 10 highly enriched differentially expressed genes and selected characteristic genes in each cell type were visualized (**Supplementary File 3**, **Supplementary Figure 5**). The correlation heatmaps of different cell types showed that the cell clustering on the scRNA-seq dataset was successful, highlighting the cellular heterogeneity of bighead carp spleen (**Supplementary File 3**, **Supplementary Figure 6**). These annotations enabled quantitative comparisons of infection driven compositional shifts and identification of the cell types driving *mapk6*/*mapk11* induction.

### 3.8 Infection remodels myeloid composition and activates a coupled IFN–MAPK program centered on mapk6/11

**Figure 4:**
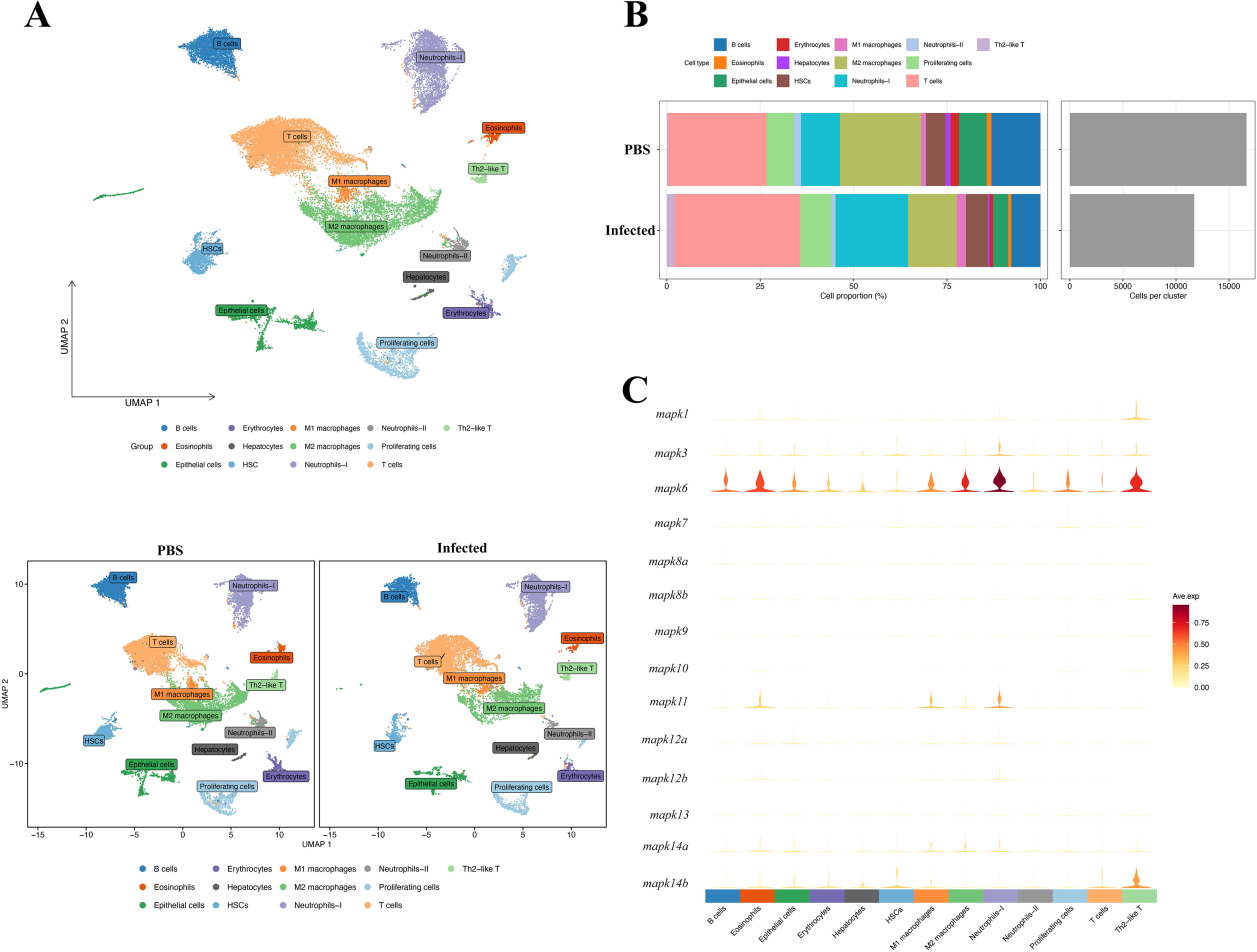
Single-cell landscape of spleen at 3 dpi and *mapks* expression across 13 cell types. **(A)** UMAP of all cells and split by group showing annotated populations. **(B)** Stacked bars summarize cell type proportions in PBS and infected groups; right panel shows the total number of captured cells per group. **(C)** Violin plots of *mapk* family genes across cell types.

There were no differences in cell aggregation or cell annotation between the control and infected groups (**Figure 4A**). Three days after infection, the proportion of Neutrophils-I increased from 10.4% to 19.4% (**Figure 4B**). The number of Neutrophils-I was 1742 in the control group and 2274 in the infected group (**Supplementary File 1**, **Supplementary Table 10**; **Supplementary File 3**, **Supplementary Figure 7**), indicating that infectious stress significantly increased the number of Neutrophils-I (*p < 0.01*). These compositional shifts indicate that infection predominantly remodels the myeloid compartment, with Neutrophils-I showing the most pronounced expansion. Furthermore, during infection, the number of T cells, M1 macrophages, and Th2-like T cells also increased, while the number of B cells, M2 macrophages and Neutrophils-II decreased significantly (*p < 0.01*). Across cell types*, mapk3/mapk6* were broadly detected, whereas *mapk1/11/12/14* were more restricted (**Figure 4C**). Notably, *mapk11* was significantly induced in Neutrophils-I and M1 macrophages, while *mapk6* upregulation was most prominent in Neutrophils-I, pinpointing the cellular sources underlying the bulk prioritized MAPK signals.

**Figure 5:**
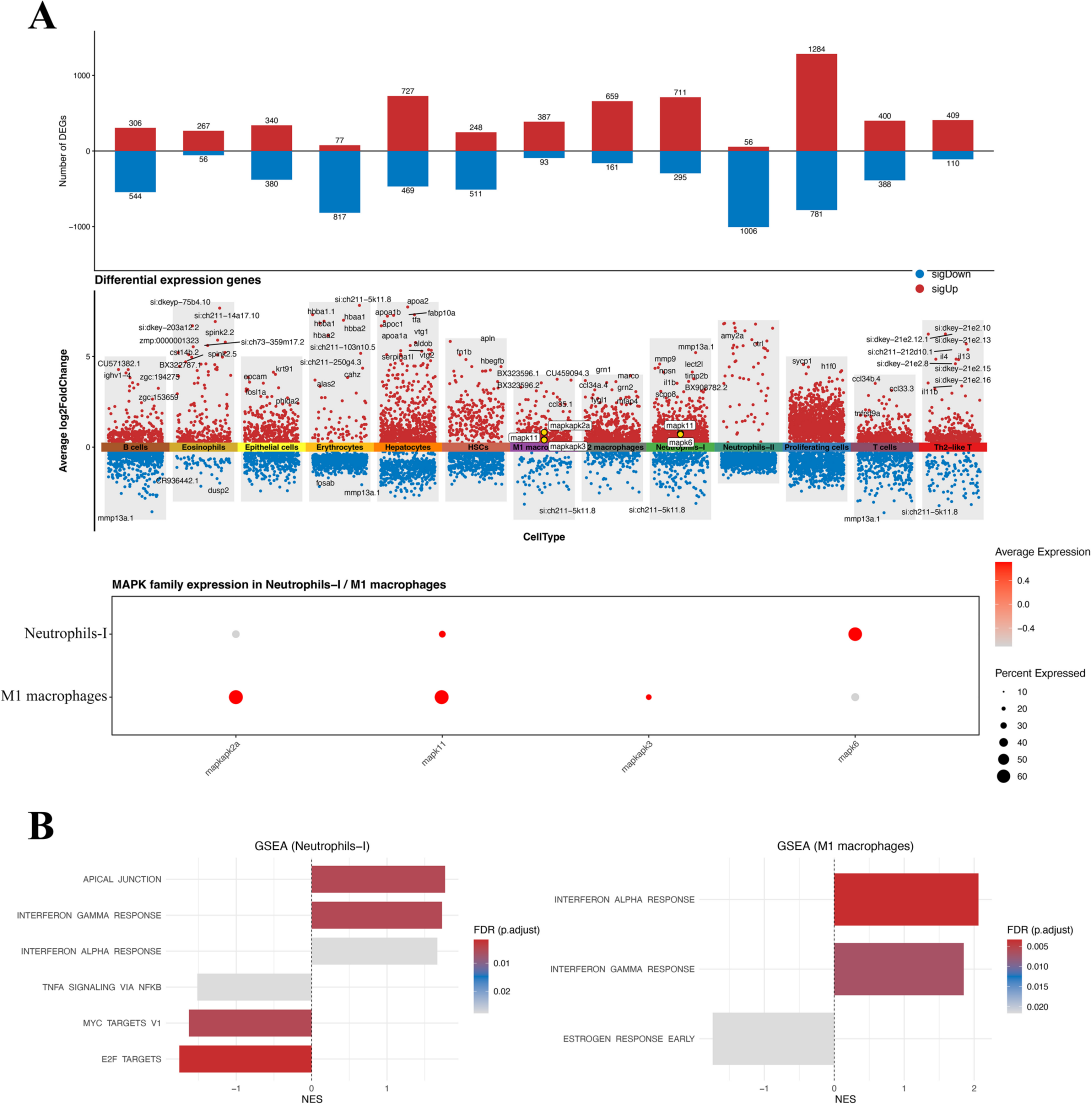
Cell-type specific differential expression and pathway signatures after infection. **(A)** Top: numbers of up- (red) and down-regulated (blue) genes per cell type. Middle: cell type resolved differential expression, with significant genes highlighted by color under the indicated thresholds. Bottom: *Mapk* family expression in Neutrophils-I and M1 macrophages. **(B)** GSEA of Neutrophils-I and M1 macrophages showing representative enriched pathways in infected group; bars indicate normalized enrichment scores (NES), shading denotes FDR.

In the differential expression analysis by cell type, the most pronounced transcriptional changes were observed in cell populations associated with proliferation and infection induced stress. A total of 2,065 DEGs were identified in Proliferating cells (1,284 up-regulated and 781 down-regulated), followed by Neutrophils-I (1,006 DEGs; 711 up-regulated, 295 down-regulated), Neutrophils-II (1,075), M2 macrophages (1,062), T cells (788), and M1 macrophages (480) (**Figure 5A**). Gene set enrichment analysis revealed that DEGs from Neutrophils-I and M1 macrophages were predominantly enriched in interferon-related signaling pathways (**Figure 5B**). This enrichment pattern aligns with the observed induction of *mapk6*/*mapk11* in these myeloid subsets, supporting a coupled IFN-MAPK stress response program during infection. Further score analysis showed that in most cell types, the MAPK branch activity score and interferon-stimulated gene (ISG) score of the infected group were synergistically increased (**Supplementary File 3**, **Supplementary Figure 8A**), suggesting that the two types of responses have a consistent upregulation trend in the context of infection (**Supplementary 3**, **Supplementary Figure 8B**). To verify the linkage between interferon response and stress pathways, we examined typical MAPK negative feedback early genes *dusp1/2/6*, *fosb*, *jun*, *atf3*, and *egr1*, which showed significant differences in key cell populations (**Supplementary 3**, **Supplementary Figure 8C**), consistent with the acute stress inflammatory signaling cascade.

### 3.9 Infection rewires myeloid-centered communication and reinforces mapk6/mapk11 along on mapk6/11

**Figure 6:**
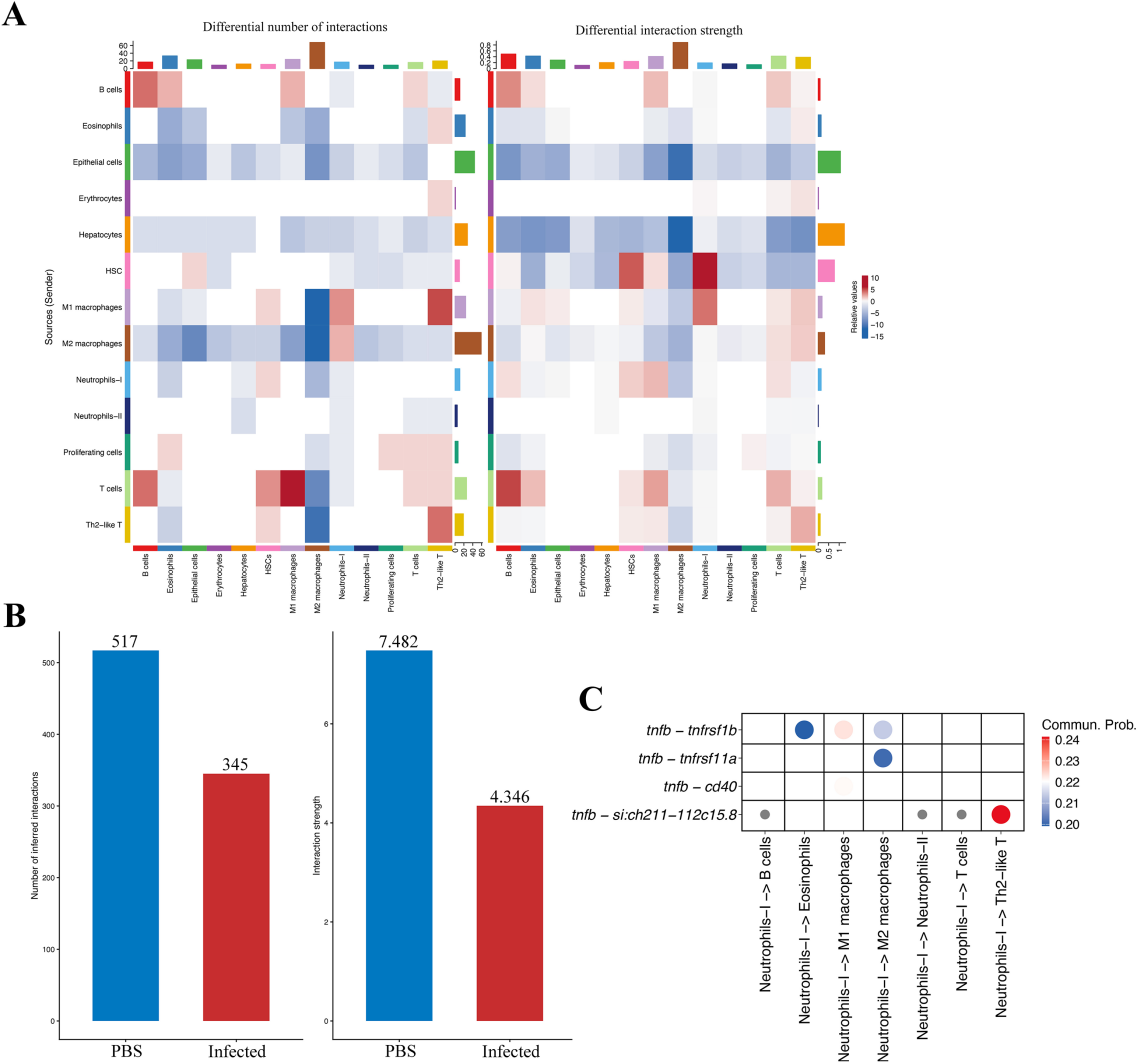
Cell-cell communication changes after infection. **(A)** Heatmaps of differential interactions. Rows indicate sender (ligand) cell types and columns indicate receiver (receptor) cell types. Left, change in the number of ligand-receptor interactions; right, change in interaction strength. Red denotes an increase in the infected group; blue denotes a decrease. Colored bars along the axes summarize row and column totals. **(B)** Summary of total interactions and overall interaction strength per group. Both metrics decline in the infected group relative to PBS. **(C)** Bubble plot of Neutrophils-I as senders to the indicated targets, highlighting representative TNFb centered ligand-receptor pairs. Dot color reflects communication probability (higher from light to red); dot size indicates significance (larger = higher −log10 P). Rows with no detected communication were removed.

This study detailed the number and strength of interactions between different cell types under infected stress (**Figure 6A**; **Supplementary 3**, **Supplementary Figure 9**). Rather than a uniform attenuation, infection caused a rewiring of intercellular communication toward myeloid centered signaling, consistent with the activation of Neutrophils-I and M1 macrophages. As shown in the heatmap (**Figure 6A**), the number of interactions between M1 macrophages and T cells, B cells, neutrophils, or Th2-like T cells increased, enhancing cellular communication. In contrast, the strength of interactions between M1 macrophages and M2 macrophages decreased, reducing communication intensity. Next, after infection, the total number of cellular interactions across 13 cell types decreased from 517 to 345, and the total cellular interaction strength decreased from 7.482 to 4.346 (**Figure 6B**). Together, these results suggest that 3 dpi of infection stress reduces intercellular communication. Additionally, the probability of ligand-receptor pairs participating in communication between neutrophils and other immune cells differed (**Figure 6C**). Specifically, there are four ligand-receptor pairs involved in communication between Neutrophils-I and other immune cells. Among them, the ligand-receptor pair tnfb-tnfrsf1b is more likely to communicate between Neutrophils-I and M1 macrophages, suggesting a key role in the immune process. This TNF axis provides a plausible communication route linking activated Neutrophils-I to macrophage activation during infection. Finally, after infection, the probability of communication between Neutrophils-I and Epithelial cells or Proliferating cells decreased, while the probability of communication between Neutrophils-I and M1 macrophages increased.

**Figure 7:**
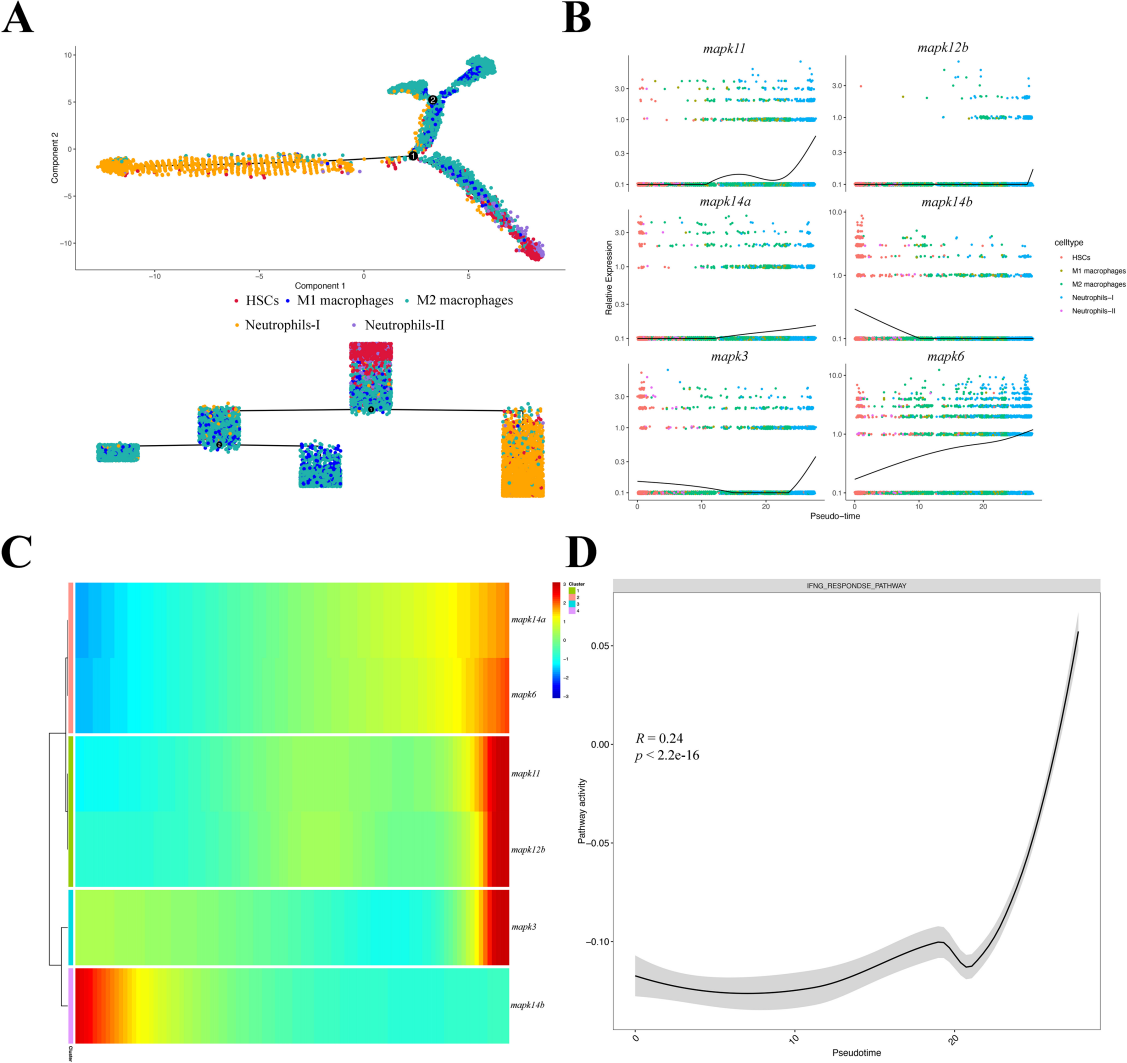
Pseudotemporal dynamics of myeloid lineages and *mapk* expression. **(A)** Single-cell trajectory illustrating transitions from HSCs toward Neutrophils-I/II and M1/M2 macrophages. **(B)** Smoothed expression of *mapks* along pseudotime; points are cells colored by identity; black curves indicate fitted trends. **(C)** Pseudotime ordered heatmap showing phase specific *mapk* dynamics. **(D)** IFN response pathway activity across pseudotime with correlation coefficient (R) and *p* value; shading denotes 95% confidence interval.

Using HSCs as the starting point for cell development, UMAP downscaling analysis constructed the cell developmental trajectories of neutrophils and macrophages, identifying two developmental decision points (**Figure 7A**). Next, during cell evolution, the expression of *mapk6* and *mapk11* genes increased significantly over time (**Figure 7B**). Consistent with their cell type specific induction, *mapk6* showed a stronger increase along the neutrophil trajectory, whereas *mapk11* increased along both neutrophil and macrophage trajectories, reinforcing their roles as key infection associated *mapks* at single-cell resolution. Notably, the expression of these two genes significantly influenced the pseudo-time trajectories, albeit with different patterns of increase (**Figure 7C**). Furthermore, enrichment of *mapk* genes with significant influence on cell evolution revealed a significant increase in the IFN pathway as HSCs develop into immune cells (**Figure 7D**). Together, these single-cell results close the loop with the bulk analyses by demonstrating that the infection associated *mapk* candidates are driven by activated myeloid subsets and are coupled with IFN programs and TNF-mediated crosstalk during infection.

## 4 Discussion

Although genome-wide analyses of the *mapk* gene family have been conducted in several fish species (15, 16, 20), the composition and evolution of *mapks* in the bighead carp remain insufficiently investigated. Based on a phylogenetic framework constructed from 382 *mapk* sequences across 24 species in our compilation, we identified 15 *Hnmapk* sequences in the bighead carp genome. These sequences clearly align with the three major subfamilies typical of vertebrates (ERK, JNK, and p38), indicating that bighead carp broadly adhere to the conserved *mapk* classification pattern. Integrating comparative genomics with tissue-, network-, and single-cell transcriptomics, we further show that bacterial challenge preferentially mobilizes a subset of family members, with *mapk6* and *mapk11* emerging as the most consistently infection associated candidates.

Further comparison with copy numbers in other vertebrates reveals that, within teleosts, the *mapk8*/*12*/*14* related branches often exhibit higher levels of retention, consistent with the FSGD experienced by fish. Considering the collinearity and copy distribution of bighead carp *mapks*, we propose that genome duplication likely constitutes the primary driver for the expansion of the *mapk*, particularly the *mapk8*/*12*/*14*. This expansion, combined with tandem or segmental duplications, collectively shaped the observed composition of *Hnmapk*. Across teleosts, copy number diversity therefore reflects a combined impact of WGD-derived retention, tandem, segmental duplication, and lineage-specific loss (13, 52), whereas despite pronounced variation in gene copy number across lineages, the *mapk* family in bighead carp maintains a conserved repertoire of functional motifs, key regulatory domains, and exon-intron organization features (13, 56). This conservation underscores that the core kinase domains are subject to rigorous functional constraints, likely due to their essential roles in signal transduction (57). Together, these patterns support a “structurally constrained but regulatory-flexible” trajectory, providing an evolutionary backdrop for context dependent recruitment of specific MAPKs during immune challenge.

In tissue level expression studies, transcriptional signals were detectable for most *Hnmapk* members, with *mapk6*, *mapk11*, *mapk12*, and *mapk14* being more highly expressed in immune-related organs (spleen and head kidney) relative to non-immune tissues. Previous studies have shown that fishes often show a MAPK pathway response after pathogen stimulation. In Japanese flounder, *ERK* and *p38* different expressed after *Edwardsiella tarda* challenge (20); *p38* up-regulation in bluntnose seabream after *A. hydrophila* infection and LPS stimulation (58), supporting MAPK involvement in antibacterial responses across teleosts. In this context, we observed a consistent upregulation of *Hnmapk6* and *Hnmapk11* in the bighead carp *A. hydrophila* infection model and obtained directionally consistent replicates in an external dataset. WGCNA provided a systems level complement to DEG based inference by placing multiple *mapk* genes within co-expression modules correlated with infection status, and by prioritizing *mapk6* within the most positively infection associated module. It is important to emphasize that these transcriptional layer trends complement rather than simply duplicate the literature on classical *mapk1/3* and *mapk14 (*14, 15), suggesting that within the *mapk* family only some of its members, particularly those highly expressed in immune organs are preferentially mobilized in response to bacterial challenge. Subsequent validation at the protein and phosphorylation level is still needed to elucidate the mechanism.

In the integrated framework spanning tissue and single cell level evidence, *mapk6* and *mapk11* appeared to be selectively mobilized in immune related cell populations following infection. ScRNA-seq of spleen revealed a bacterial response composition characterized by increased proportions of Neutrophil-I, M1 macrophages, and T cells, with relative decreases in other immune cells; this pattern is consistent with prior reports in zebrafish under pathogen challenge that describe expansion of myeloid effector compartments and reinforcement of IFN programs (59, 60). Functionally, the elevated Neutrophil-I and M1 macrophage subsets showed significant enrichment for IFN-α/γ and inflammatory antibacterial pathways, indicating activation of an innate defense axis centered on IFN and classical inflammation in bighead carp during *A*. *hydrophila* exposure. Crucially, the single-cell data closed the loop on bulk prioritization by localizing gene induction to specific effector populations, *mapk11* was significantly upregulated in Neutrophils-I and M1 macrophages, whereas *mapk6* induction was most prominent in Neutrophils-I. This pattern suggests that infection responsive MAPK signaling is preferentially coordinated within the myeloid compartment rather than uniformly activated across all leukocytes, which is consistent with the central roles of neutrophils and macrophages in teleost antibacterial immunity (61). In vertebrates, TNF receptor signaling can engage MAPK branches and thereby modulate macrophage inflammatory activation, suggesting that the concurrent enrichment of *mapk6*/*mapk11* and IFN-related pathways in Neutrophils-I and M1 macrophages may reflect a cooperative myeloid regulatory program during early infection (62).

CellChat analysis further indicated enhanced interactions between M1 macrophages and Neutrophil-I after infection, whereas global interaction number and strength decreased, suggesting network reorganization rather than synchronous amplification of all outward ligand receptor signals (63). In line with this interpretation, receptor-ligand usage was dominated by tnfb-tnfrsf1b, while IFN-related genes were predominantly upregulated intracellularly, pointing to a response primarily driven by intracellular anti-infective programs. Given that TNF-family cytokines are conserved activators of fish phagocytes and macrophages (64), the tnfb-tnfrsf1b axis may represent a local signal through which activated Neutrophils-I promote the activation or maintenance of neighboring M1 macrophages, although this will require direct functional validation. These observations support a model in which a local TNF-linked axis accompanies a predominantly cell intrinsic IFN program during early–mid infection stages (65). Notably, *mapk6* was broadly expressed across all 13 profiled cell types, and *mapk3*/*11*/*14a* increased along pseudotime during HSCs differentiation toward M1 macrophage and Neutrophil-I lineages. These single-cell observations converge with our bulk and WGCNA findings, namely infection induced expression and higher baseline abundance in immune organs and support preferential recruitment of specific family members rather than uniform activation of the *mapk* family. Within an evolutionary pathway of copy number variability coupled with structural conservation, highly expressed *mapk* members that are coupled to immune axes appear to be prioritized in IFN enriched myeloid effector cells, thereby completing a coherent trajectory from family expansion to tissue and infection level expression and finally to single cell functional mobilization (66). Taken together, these transcriptomic data delineate preferential *mapk6*/*11* recruitment. Although *mapk6*/*11* were validated at both bulk and single-cell levels, protein- and phosphorylation-level assays are still required to establish causal pathway activation.

## 5 Conclusions

In this study, we performed a genome-wide characterization of the *mapk* gene family in bighead carp and identified 15 members that could be clearly assigned to the ERK, JNK and p38 subfamilies. By integrating gene structure, conserved motifs, synteny and phylogenetic analyses across 24 species, we confirmed that fish-specific duplication contributed to the retention of additional *mapk8/mapk12/mapk14* copies. Expression profiling, together with WGCNA, showed that several *mapk* genes, particularly *mapk6* and *mapk11*, were strongly activated in spleen and head kidney following *Aeromonas hydrophila* infection. ScRNA-seq further localized this MAPK–IFN activation to infection expanded myeloid populations and revealed TNF mediated crosstalk among immune cells. These findings provide a comprehensive framework for *mapk* evolution and immune deployment in bighead carp and supply candidate genes for functional studies and disease resistance improvement in cyprinid fishes.

## Data Availability

The public datasets analyzed in this study have been deposited in the GEO repository under accession number GSE57952.
