# Supplementary material for: Genome to single-cell characterization of the MAPK family in bighead carp (Hypophthalmichthys nobilis) spleen during Aeromonas hydrophila challenge
Source: Front Immunol. 2026 Apr 13;17:1777786. doi: 10.3389/fimmu.2026.1777786 (PMC13110963; doi:10.3389/fimmu.2026.1777786)
Supplement: Supplementary file 1 [file Table1.docx]

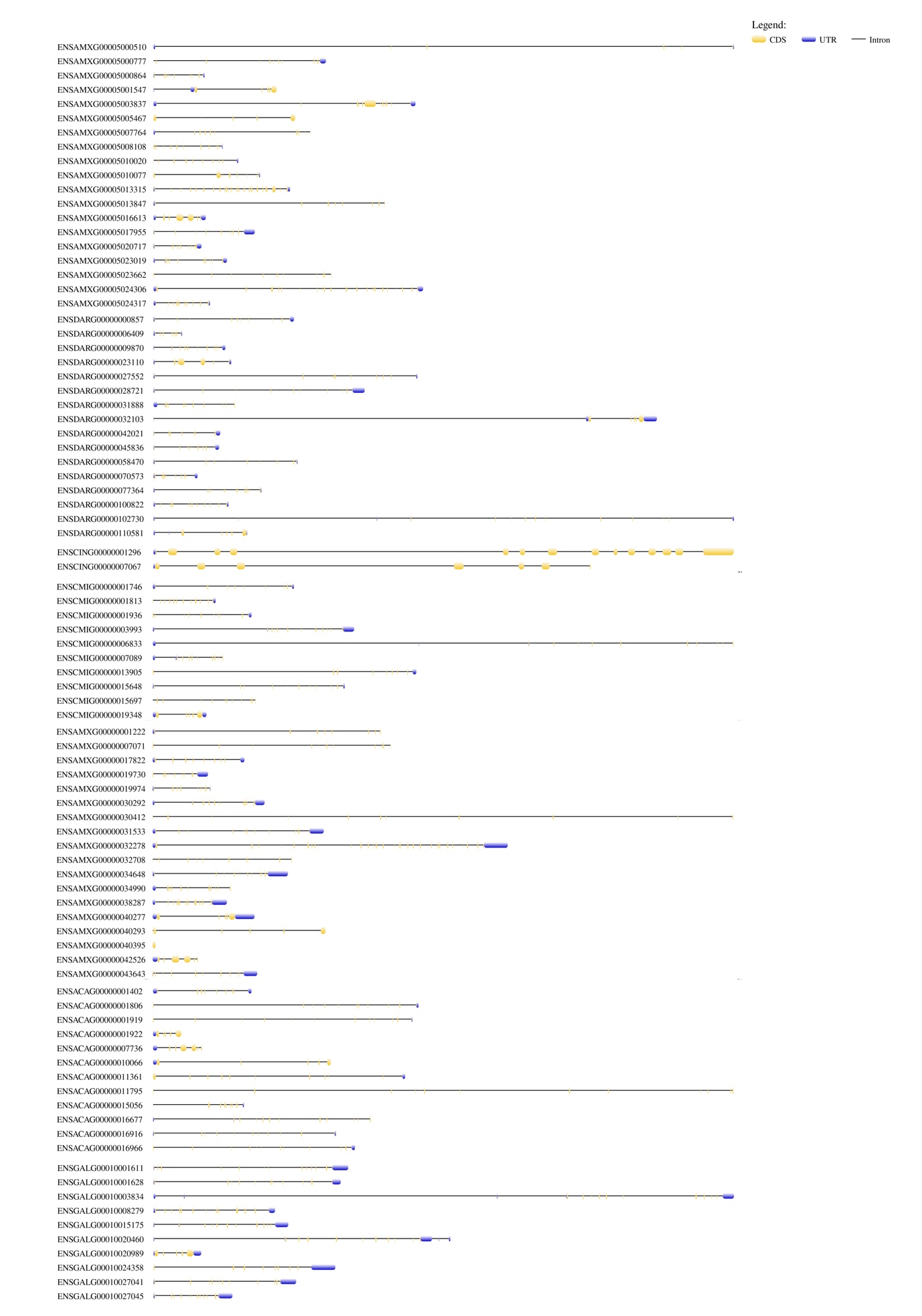

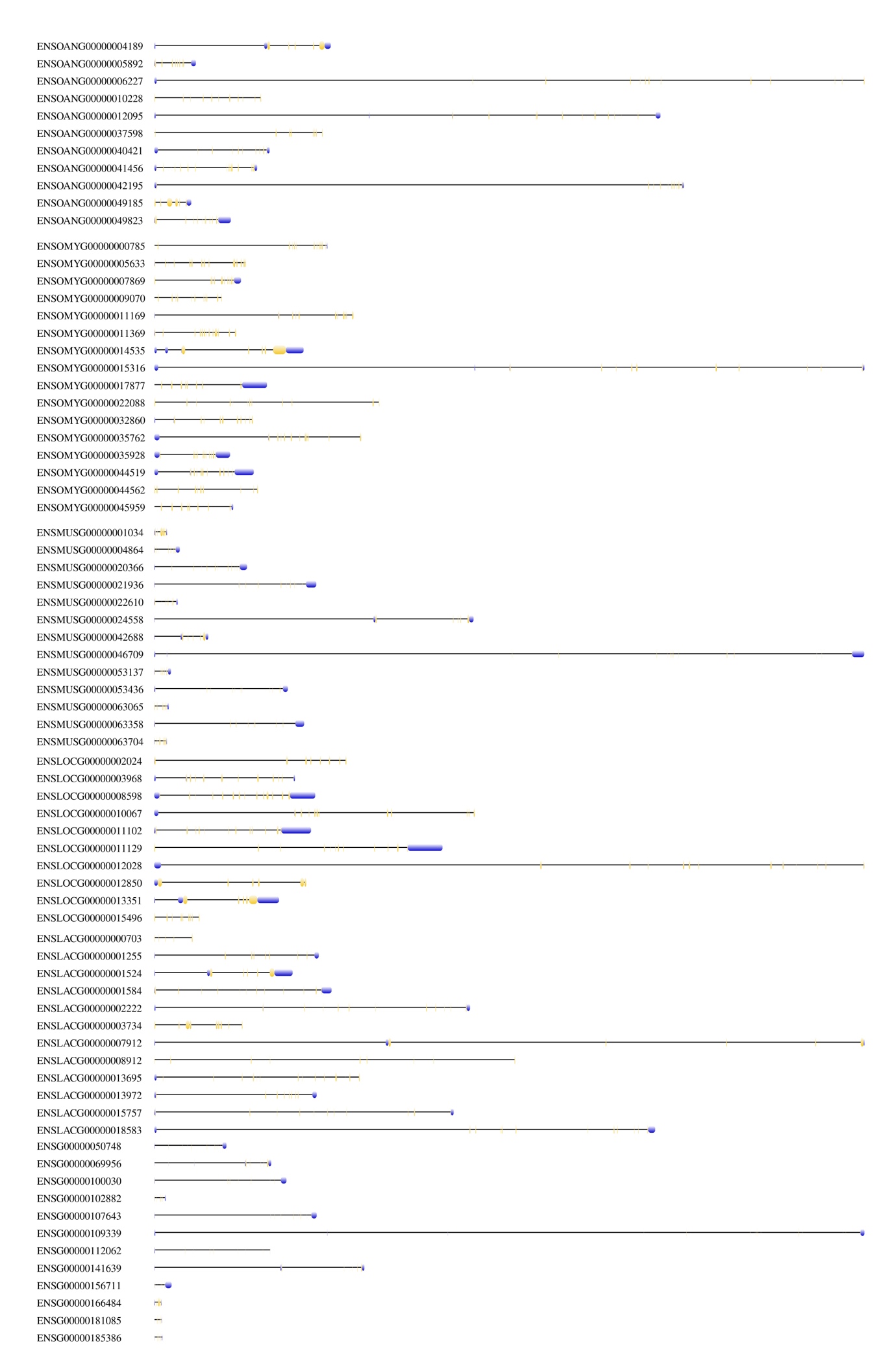

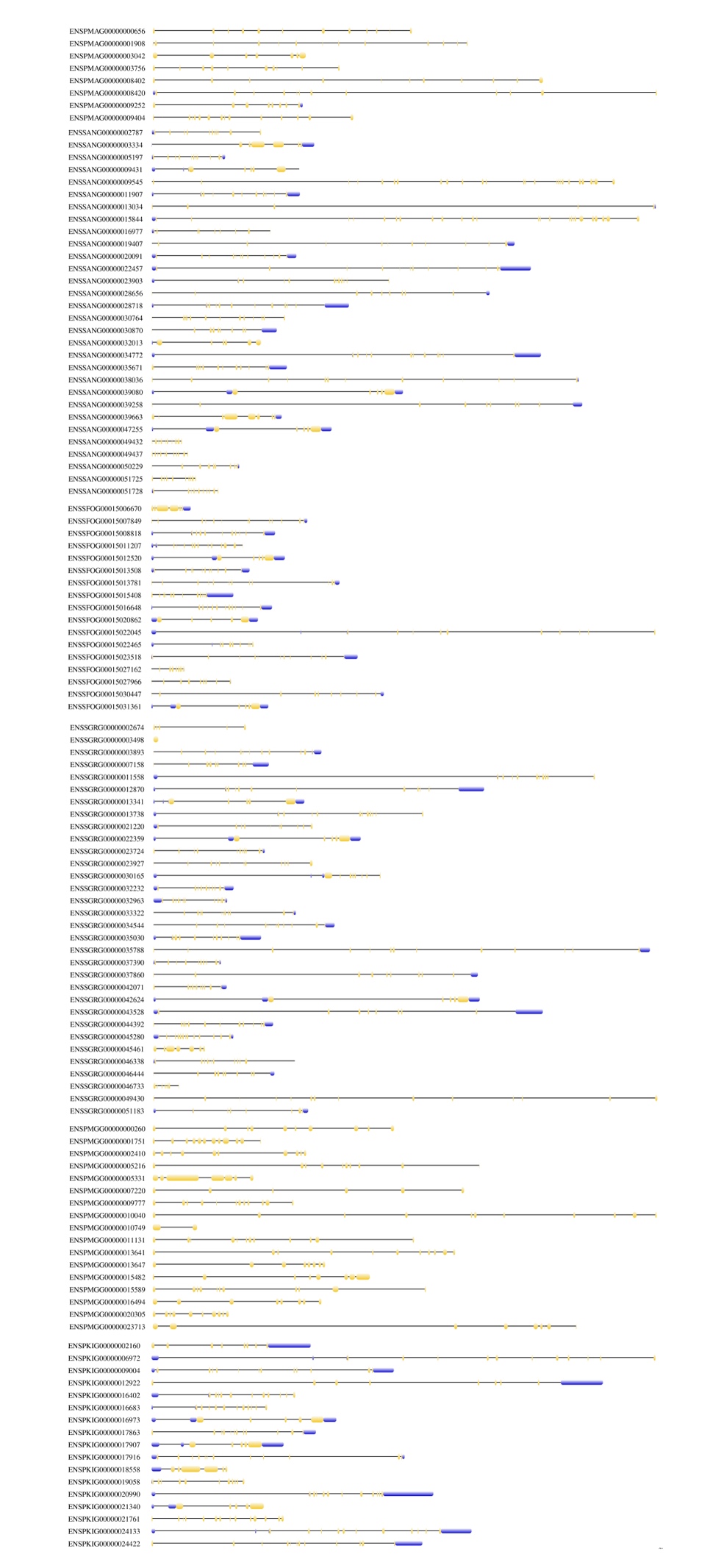

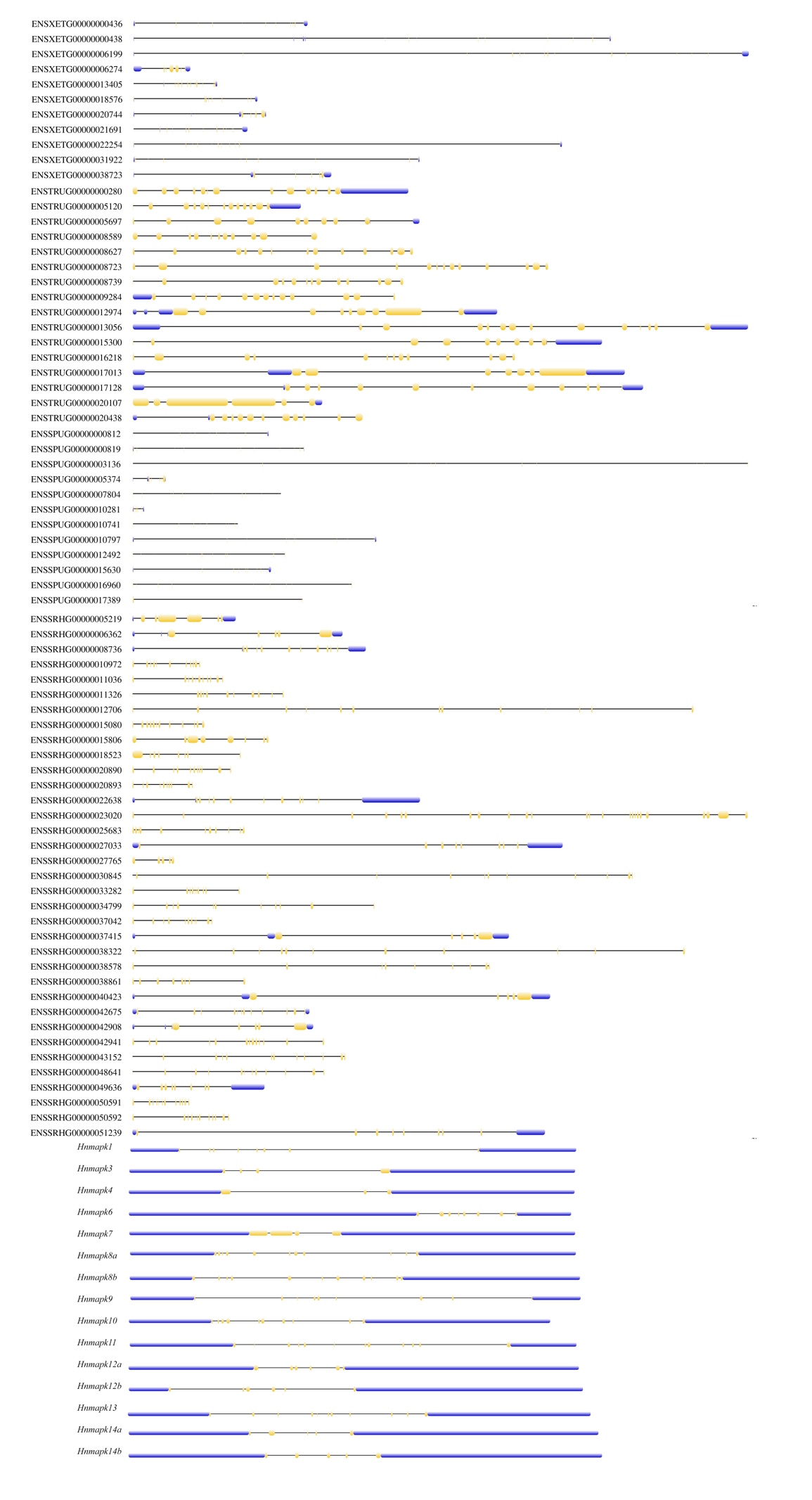


Fig. S1. Gene structure of 382 *mapk* genes from 24 species.


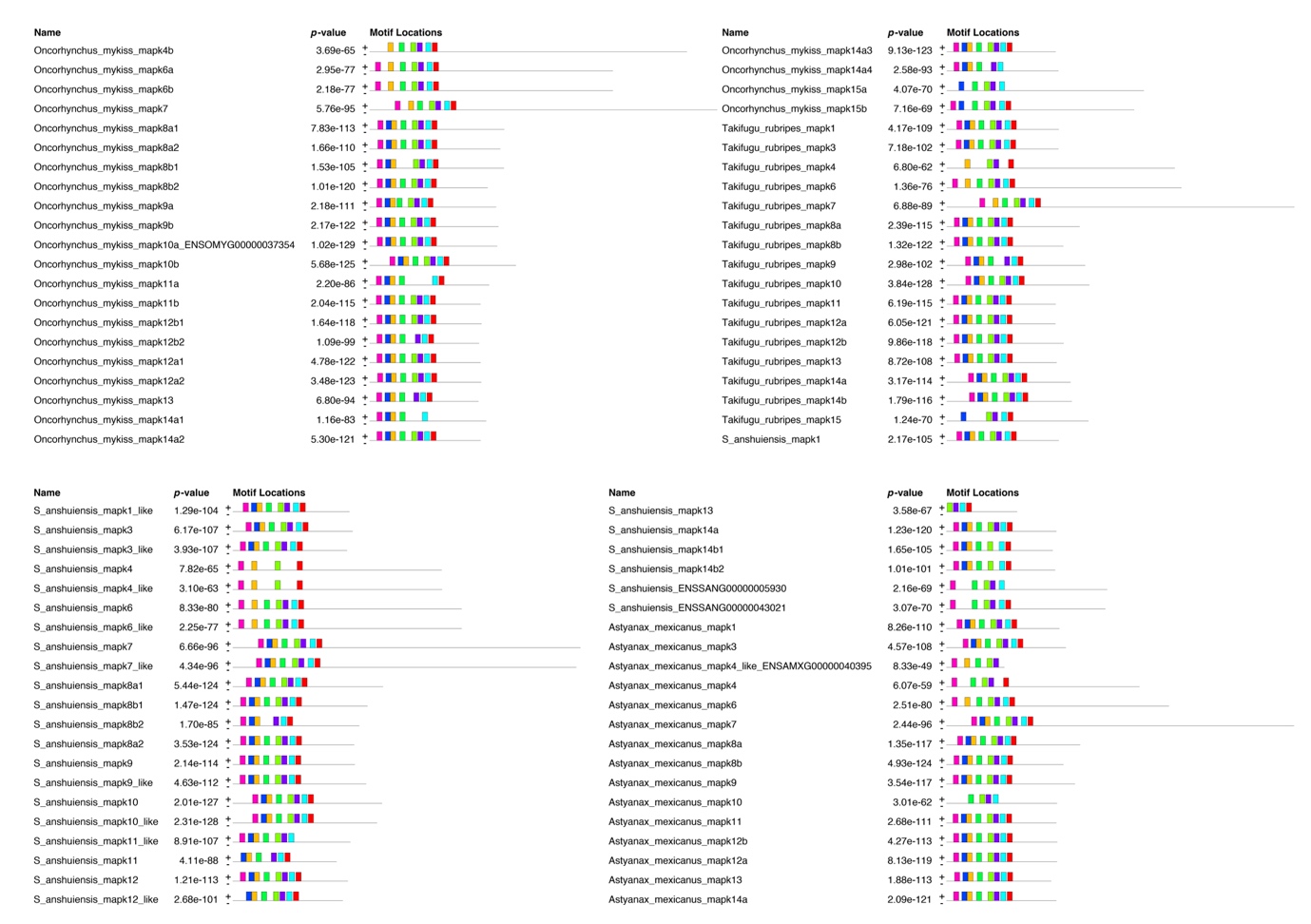

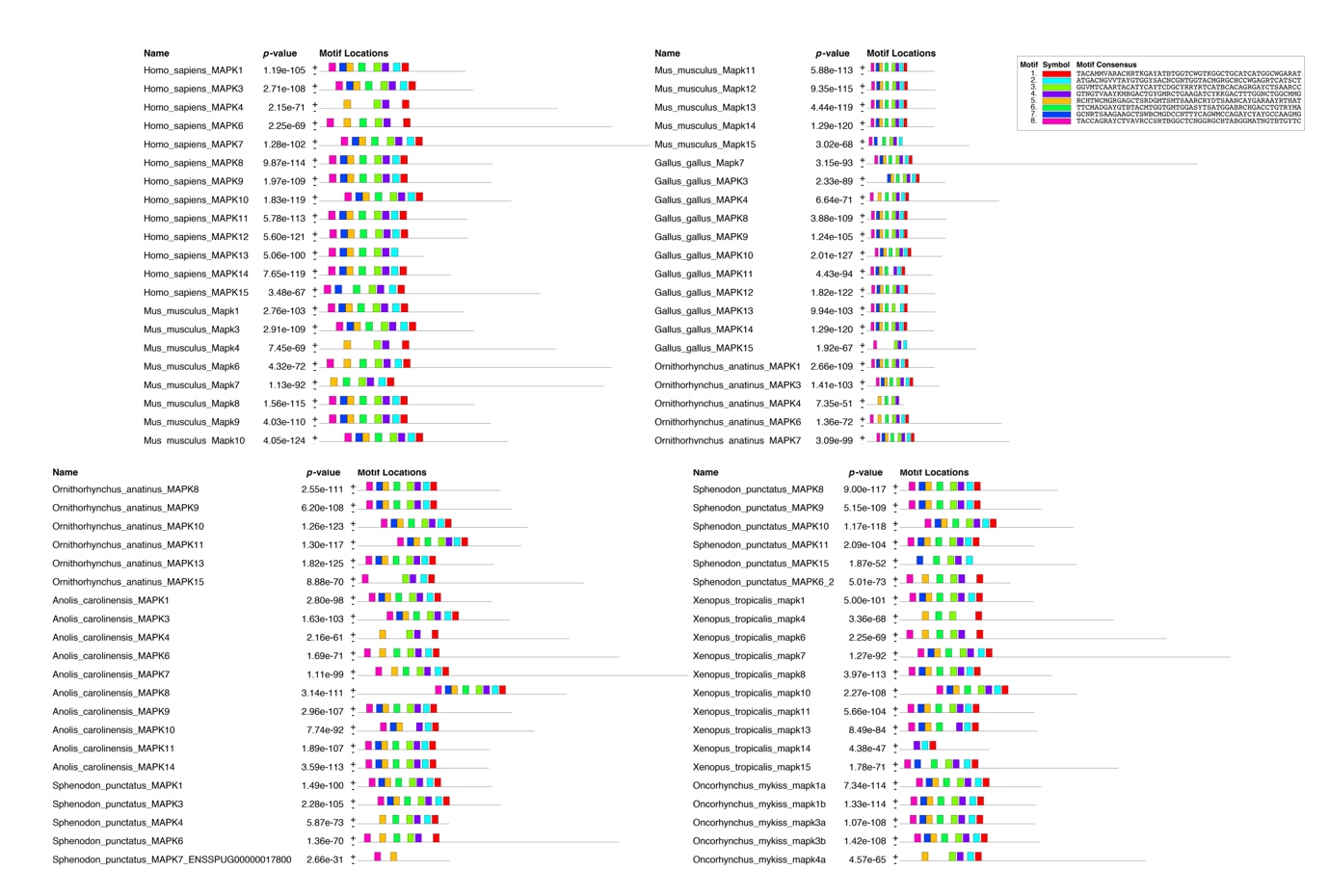

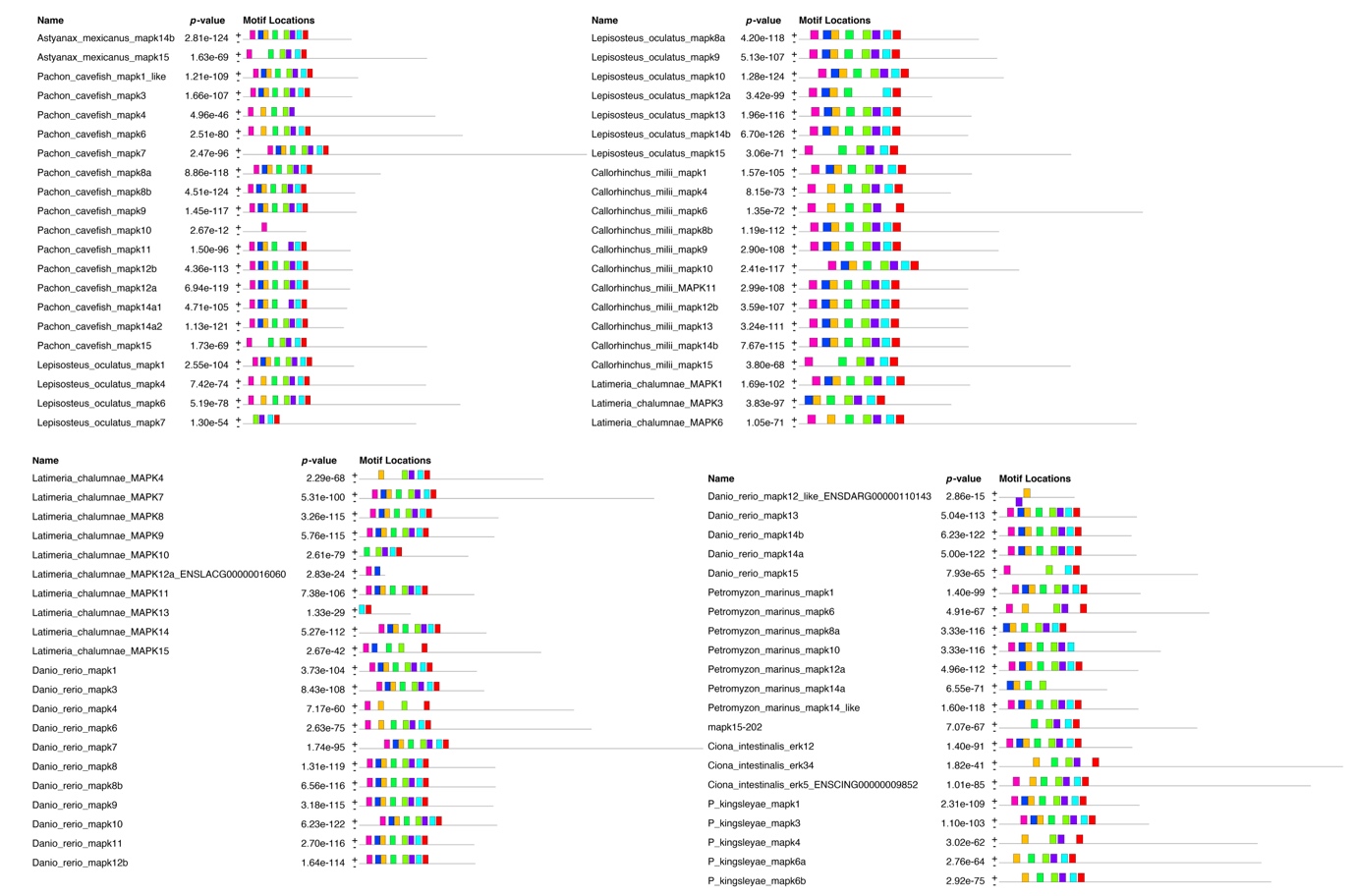

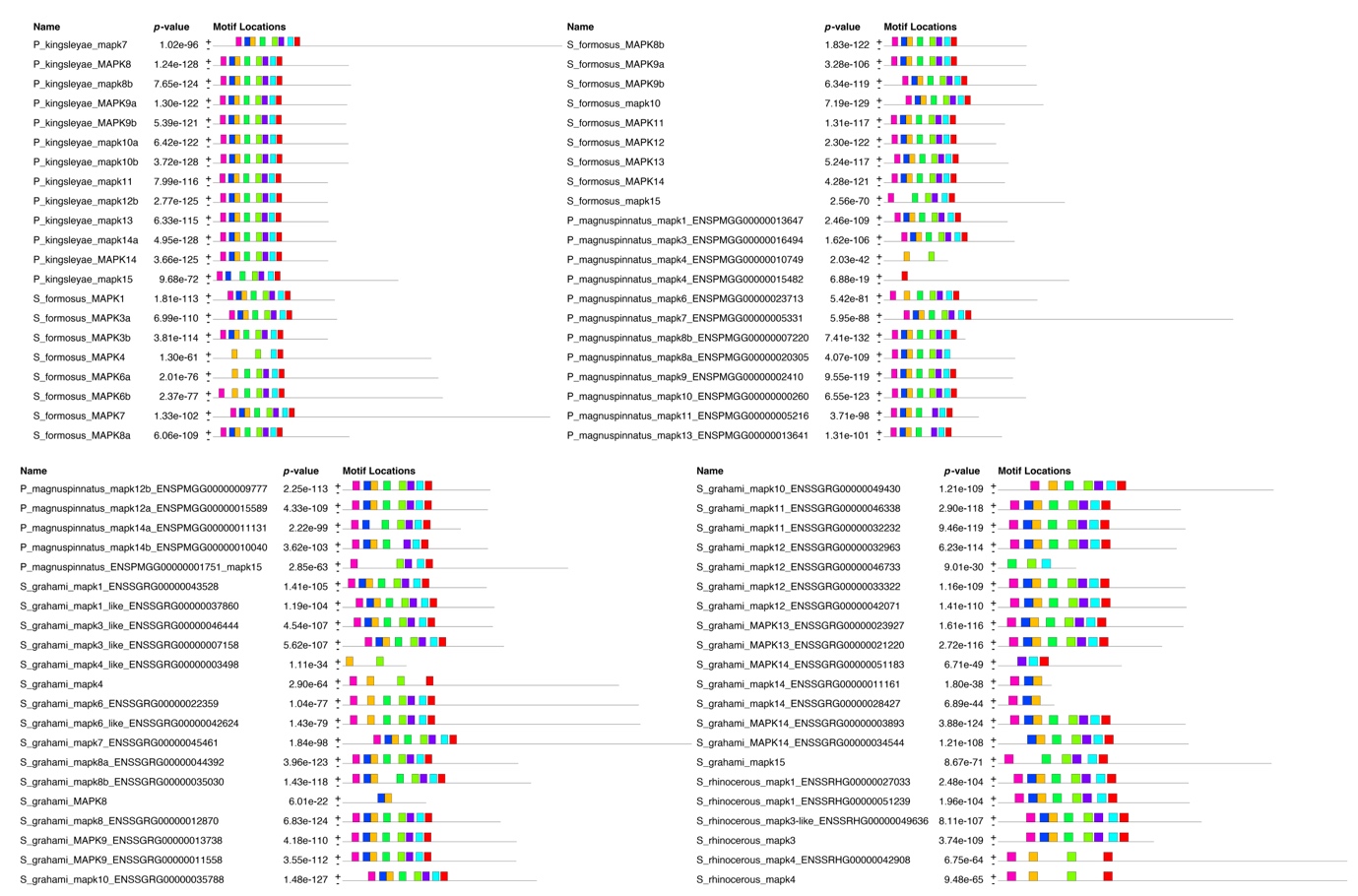

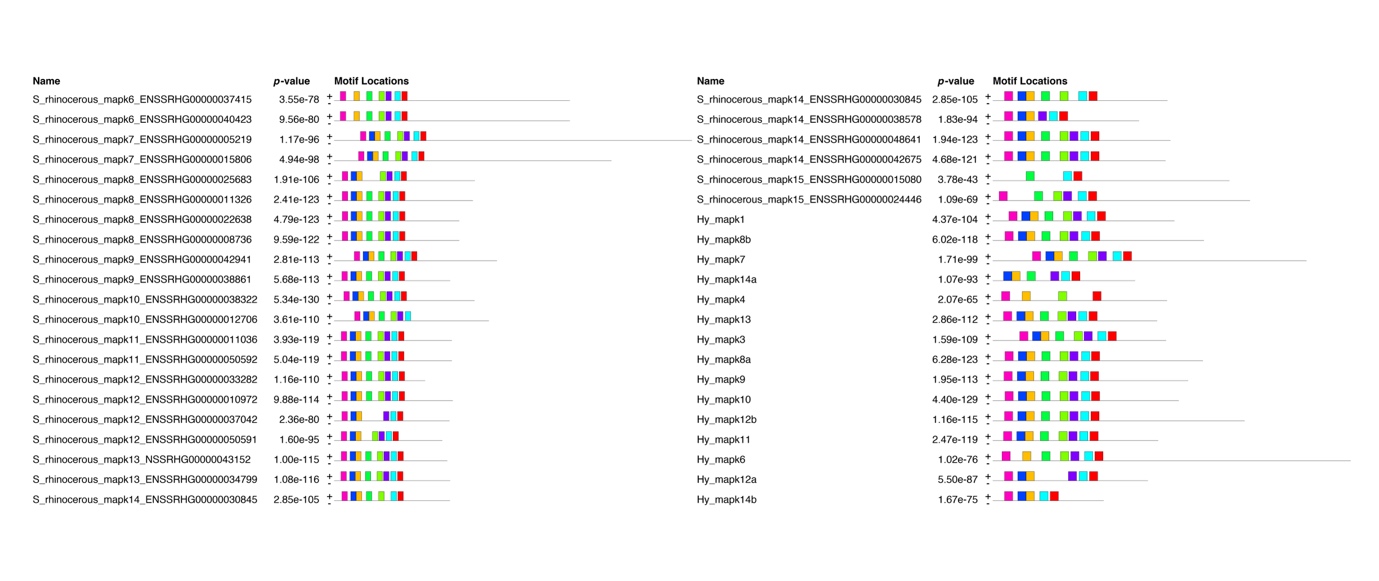


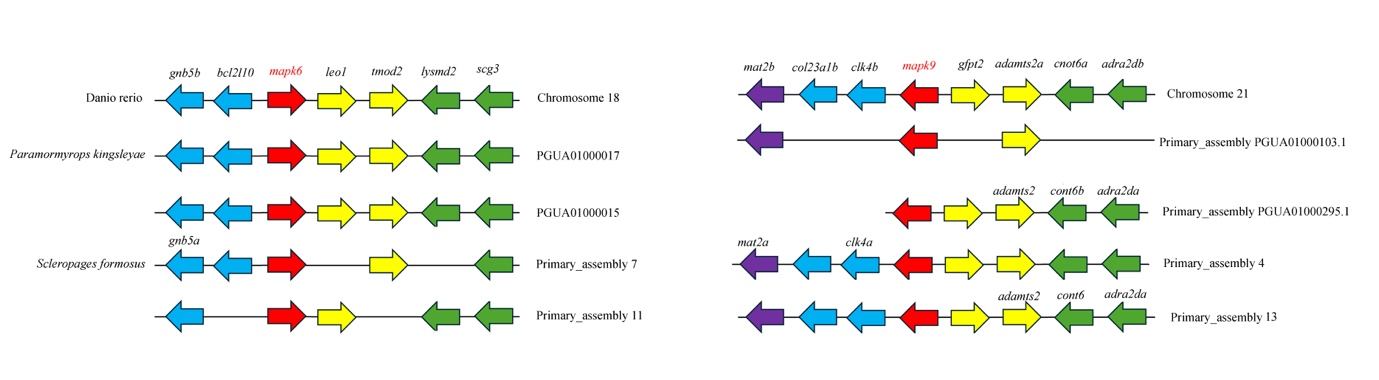


Fig. S2. Gene motifs of 166 stk genes from 27 vertebrates and mapk6/9 in *Scleropages formosus* and *Paramormyrops kingsleyae*.


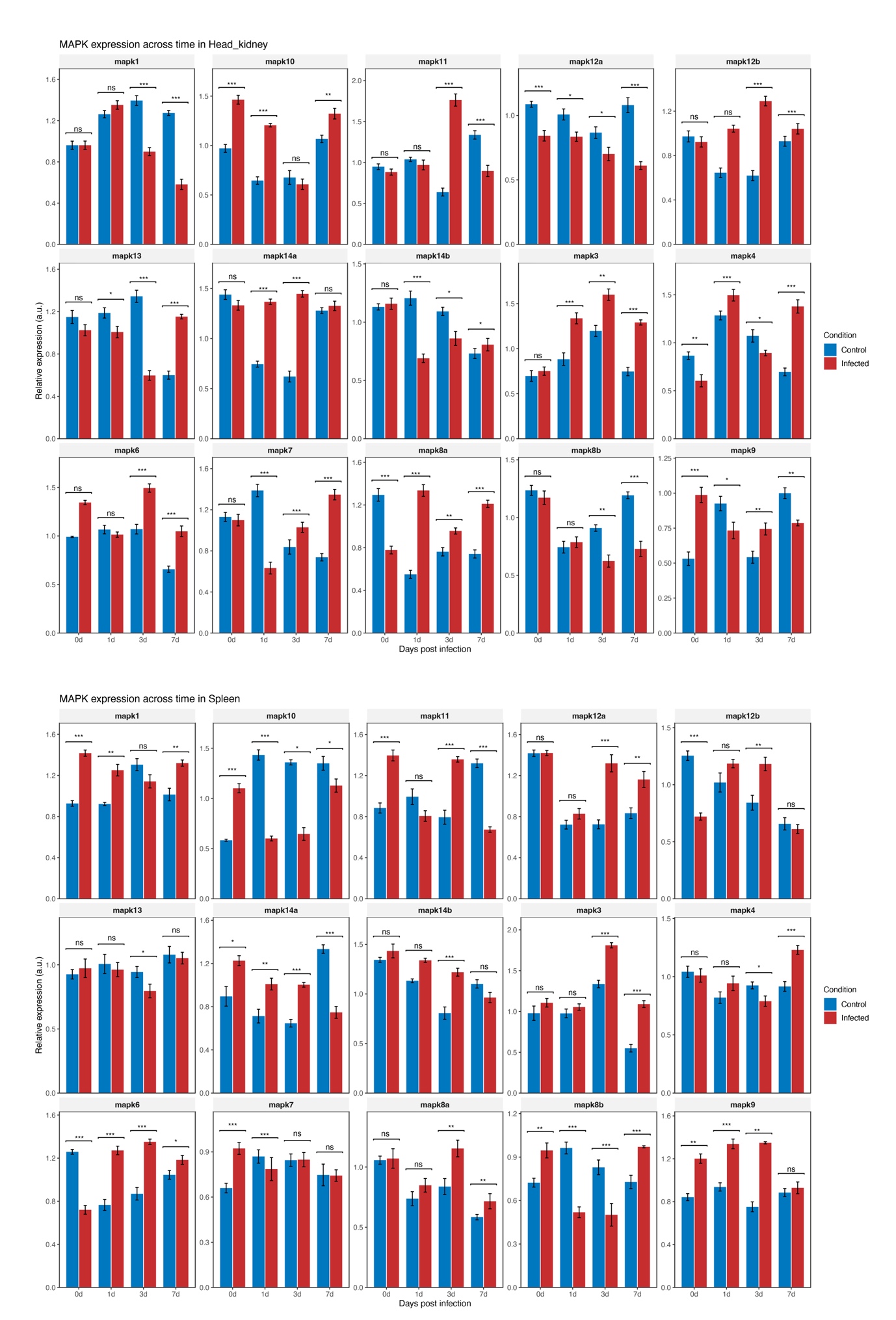


Fig. S3. *Mapks* expression analysis and its function in response to bacterial infection stressors. (A) The relative level of *mapks* expression in bighead carp head kidney at various time intervals following Aeromonas hydrophila inoculation. (B) The relative level of *mapks* expression in bighead carp spleen at various time intervals following *Aeromonas hydrophila* inoculation. *, p < 0.1; **, p < 0.05; ***, p < 0.01.


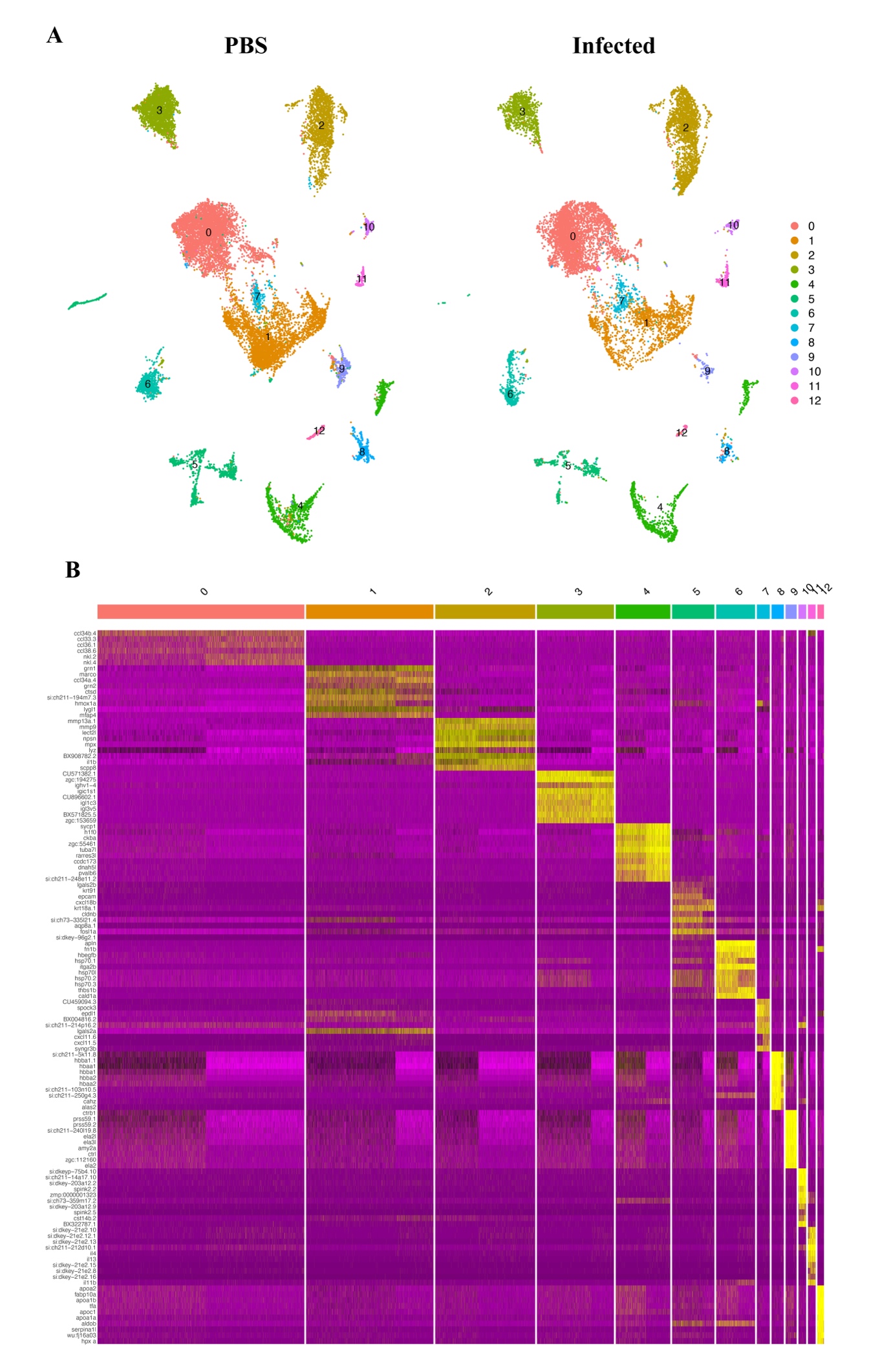


Fig. S4. (A) UMAP plot of cell clustering in control group and infected group. (B) Heatmap of gene expression in different cell clusters.


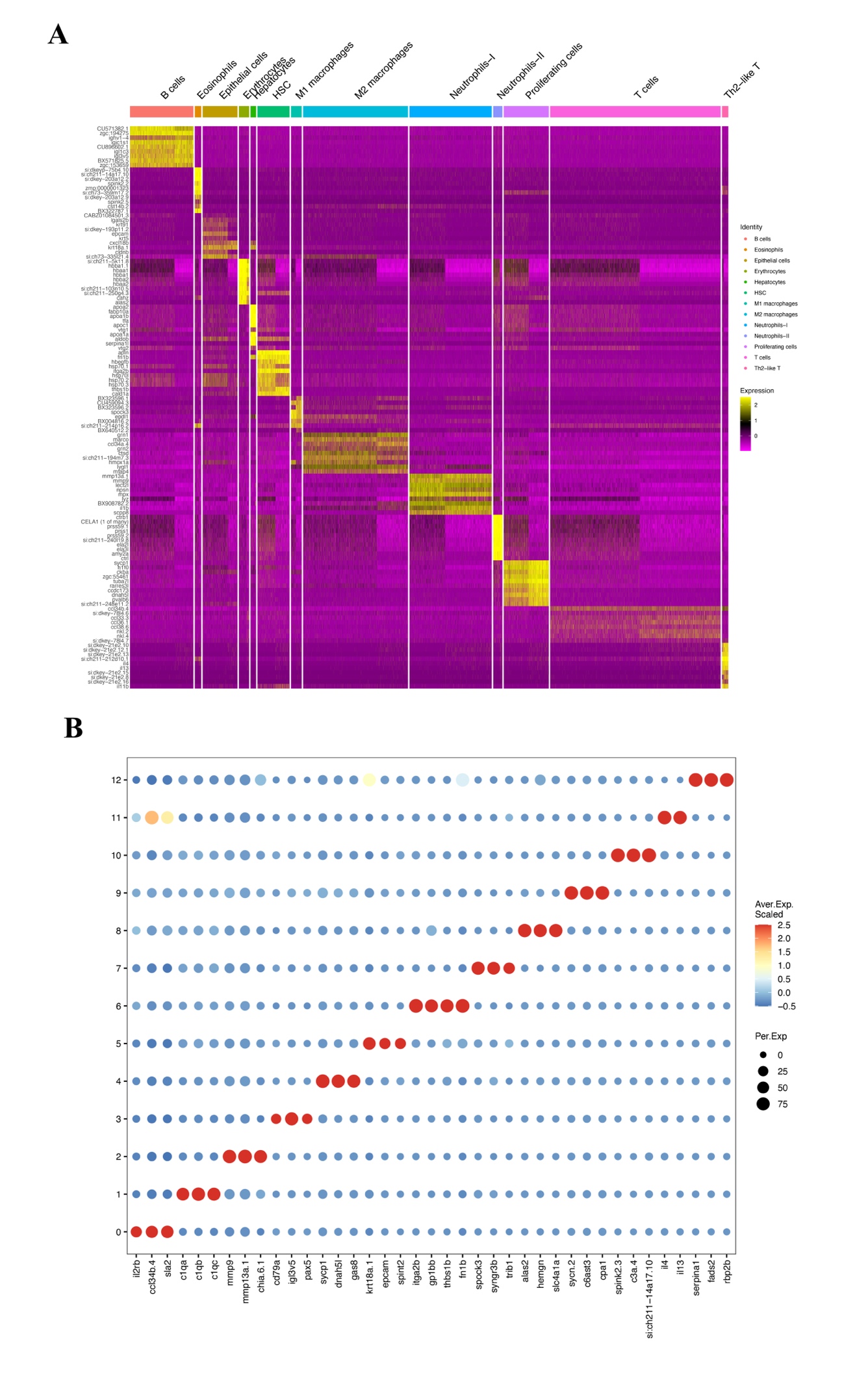


Fig. S5. (A) Heatmap of gene expression in different cell types. (B) Bubble plots of characteristic gene expression for different clusters.


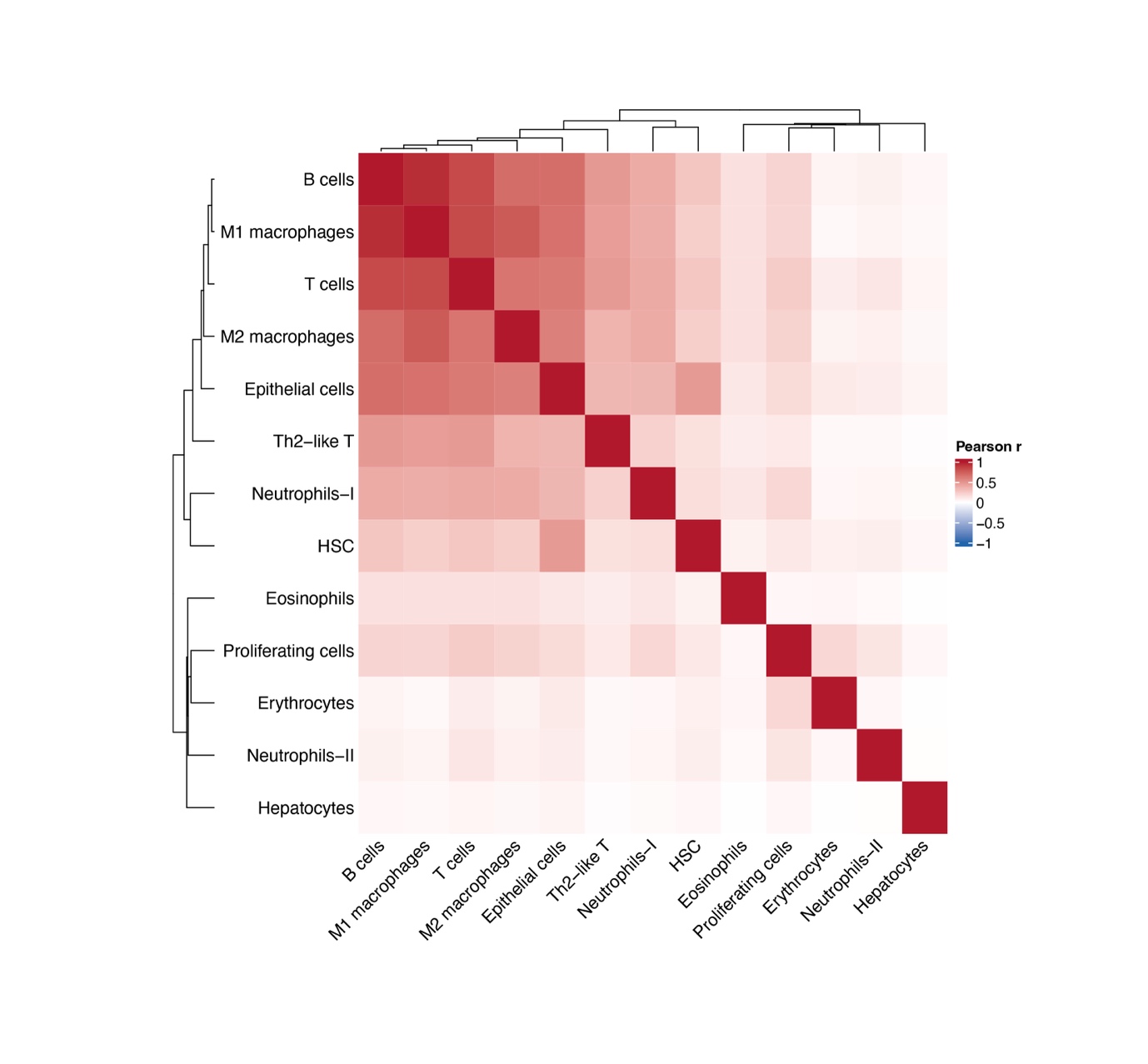


Fig. S6. Heatmap of correlations between different cell types.


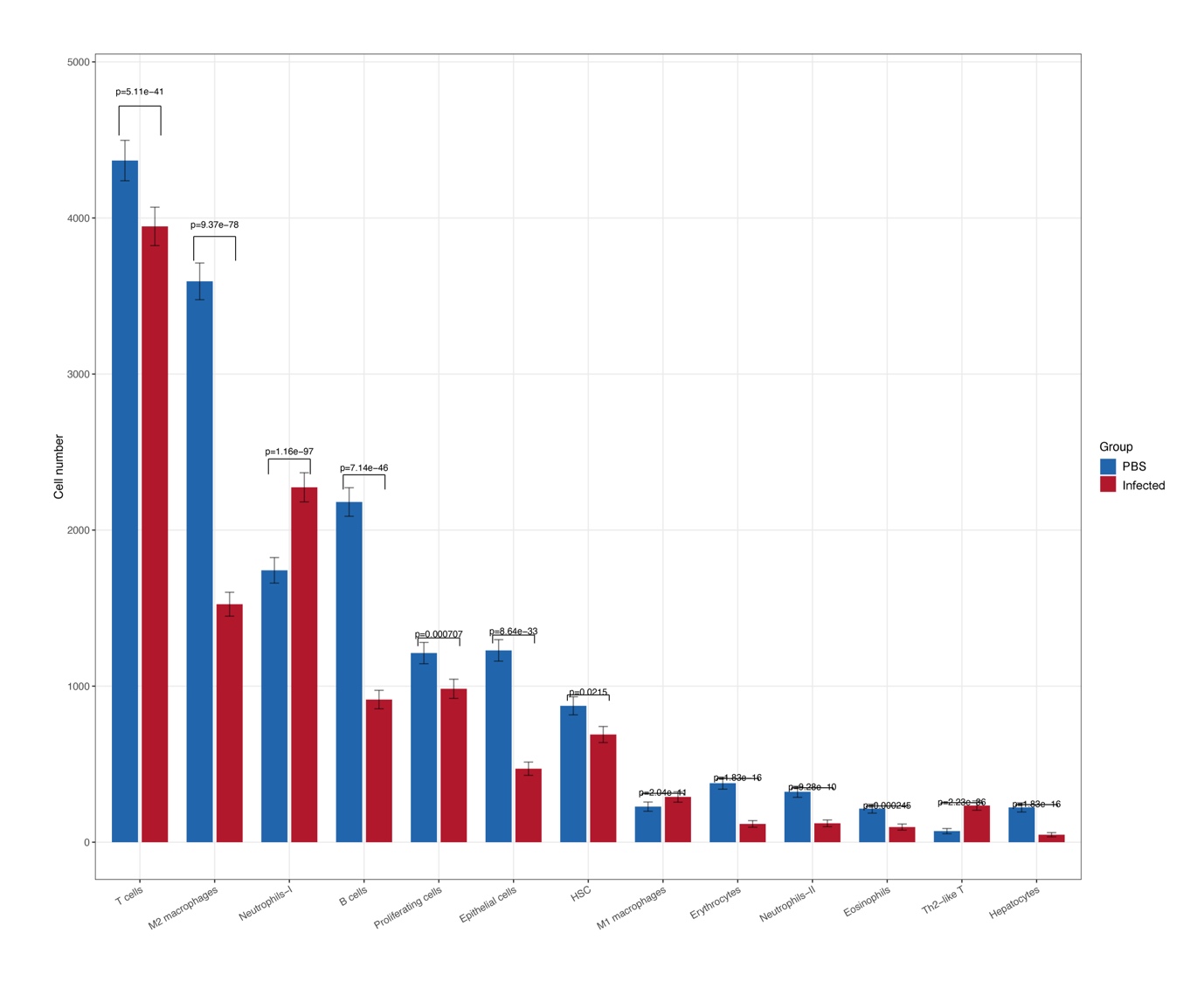


Fig. S7. Comparison of the differences in the number of cells of different cell types in the control group and infected group.


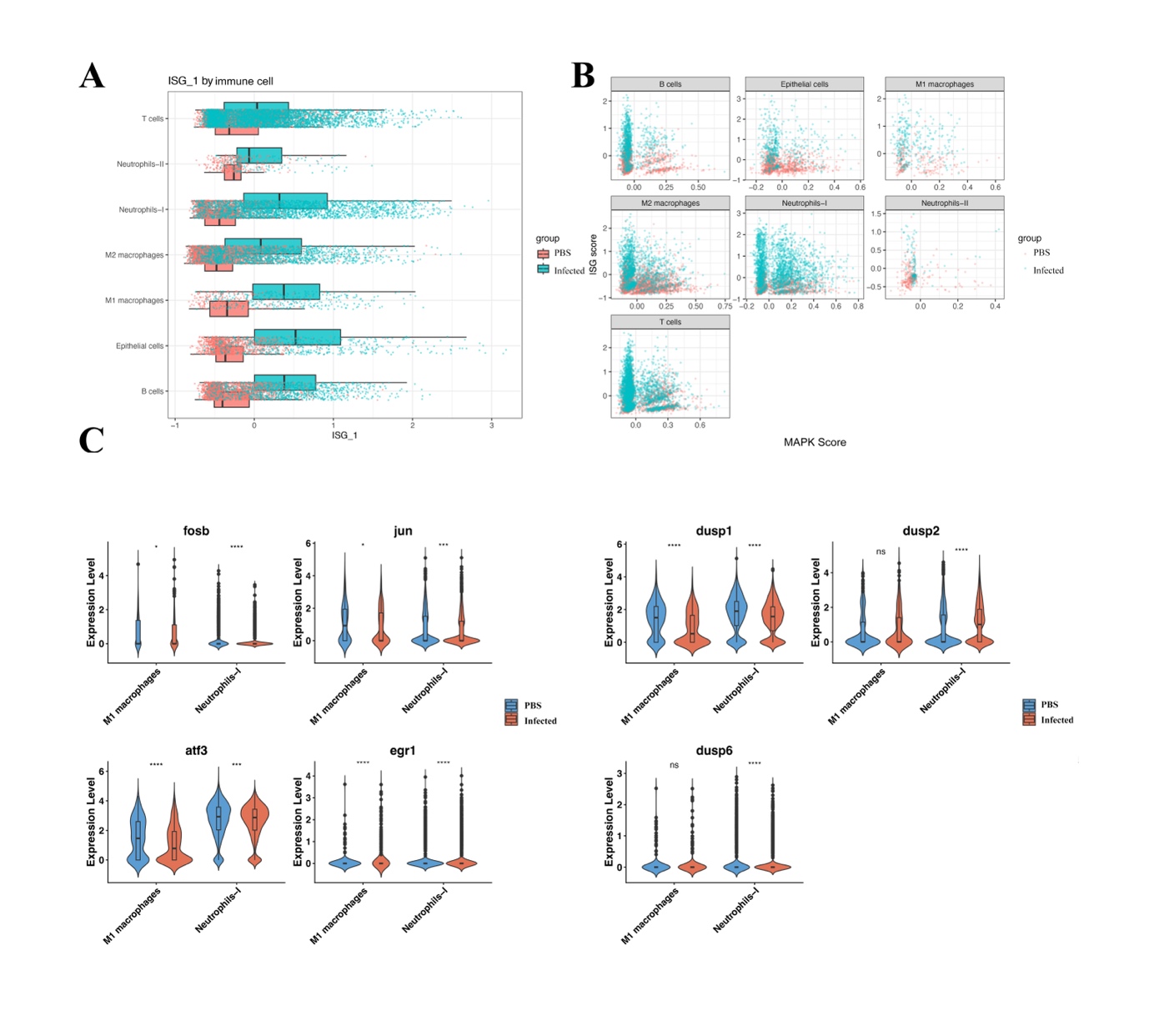


Fig. S8. Infection elevates ISG activity and links it to MAPK signaling. (A) ISG_1 scores by cell type: higher in infected cells, strongest in Neutrophils-I/II and M1 macrophages; (B) MAPK vs. ISG_2: positive coupling in M1 macrophages, Neutrophils-I/II, and T cells; weak in B and epithelial cells; (C) Gene expression in M1 macrophages and Neutrophils-I: *fosb*, *jun*, *atf3*, *egr1* and *dusp1*/*2*/6 are mostly upregulated with infection. Colors: blue = PBS, red = infected. Stats: two-sided Wilcoxon; ns not significant; P < 0.05, ** < 0.01, *** <0.001, **** <0.0001.


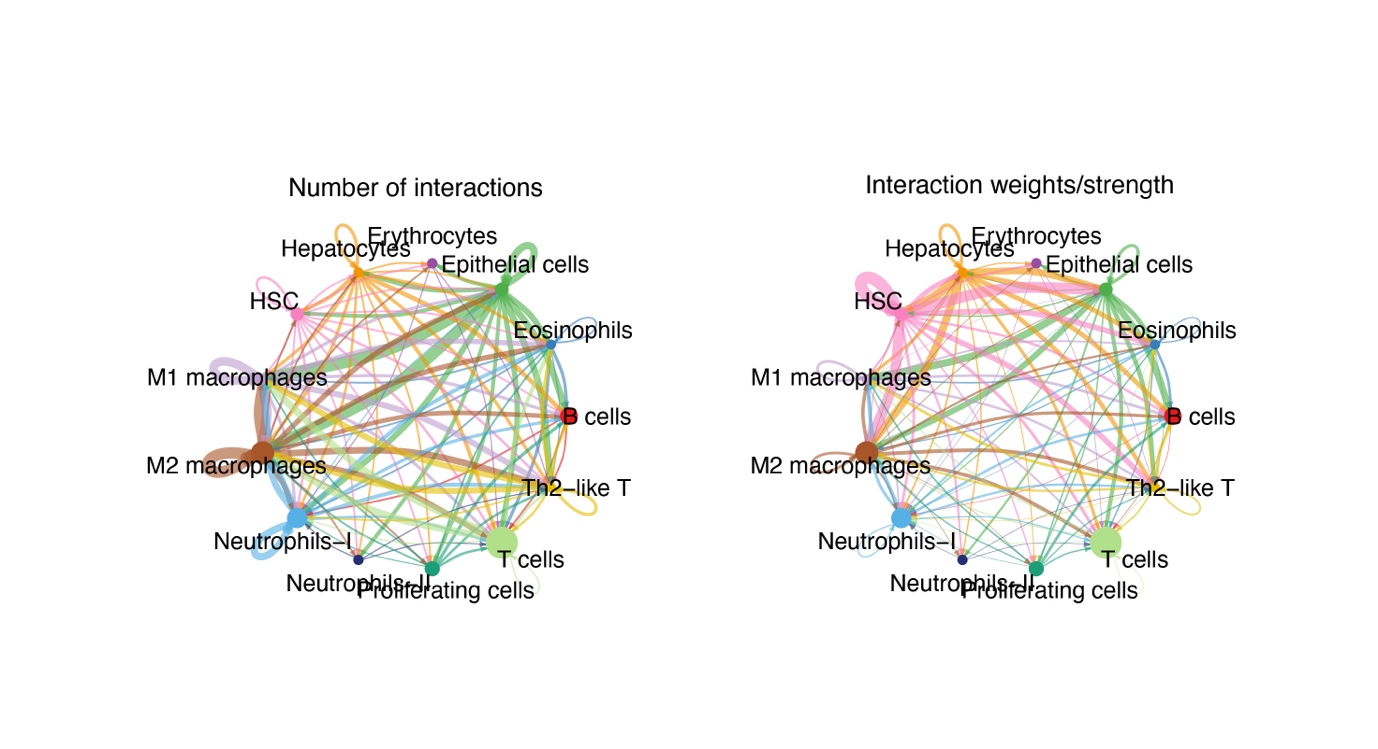


Fig. S9. Network plot of differences in cell-cell communication between control group and infected group. Blue colour represents decrease, and red colour represents enhancement.
